# Bordetellae colonization oligosaccharide (b-Cool), a glycan crucial for nasal colonization

**DOI:** 10.1126/sciadv.adw7764

**Published:** 2025-09-03

**Authors:** Yang Su, Maiya Callender, Dylan Woolsey, Joanna Zhu, Colleen Sedney, Mimi Thu Vu, Jillian Masters, Amanda Caulfield, Kalyan Dewan, Emily Gaskill, Reilly Stevens, Austin Joshua, Christopher M. Evans, John Glushka, Evgeny Vinogradov, Andrew Preston, Thomas Krunkosky, Maor Bar-Peled, Eric T. Harvill

**Affiliations:** ^1^ Department of Biochemistry and Molecular Biology, University of Georgia, Athens, GA, USA.; ^2^ Complex Carbohydrate Research Center, University of Georgia, Athens, GA, USA.; ^3^ Department of Infectious Disease, University of Georgia, Athens, GA, USA.; ^4^ University of Colorado Anschutz Medical Campus, Aurora, CO, USA.; ^5^ Research Services, Rocky Mountain Regional VA Medical Center, Aurora, CO, USA.; ^6^ National Research Council Canada, Human Health Therapeutics Centre, Ottawa, Canada.; ^7^ The Milner Centre for Evolution and the Department of Life Sciences, University of Bath, Bath, UK.; ^8^ Department of Biomedical Sciences, University of Georgia, Athens, GA, USA.; ^9^ Department of Plant Biology, University of Georgia, Athens, GA, USA.

## Abstract

Respiratory tract infections pose considerable global health challenges. Upper airway colonization is pivotal to these infections, including those caused by *Bordetella* species. We identified an oligosaccharide, bordetellae colonization oligosaccharide (b-Cool), crucial for early nasal colonization of *Bordetella bronchiseptica*. We characterized the structure of b-Cool by LC-MS and NMR and found that it is prevalent across a diverse range of bordetellae, including *Bordetella pertussis*, which causes whooping cough in humans. A *B. bronchiseptica* mutant lacking b-Cool (Δ*b-Cool*) showed significantly delayed and decreased colonization in mouse nasopharynx and nasal epithelia, resulting in decreased transmission. The colonization defect of Δ*b-Cool* was rescued in mucin deficient mice, suggesting that b-Cool may facilitate colonization in the presence of airway mucins.

## Teaser

A glycan composed of rare sugars facilitates nasal colonization and transmission of *Bordetella*.

## INTRODUCTION

Upper airway colonization is a critical early step for bacterial pathogens to establish respiratory infections (*1*), and there is emerging evidence that bacterial pathogens use glycans in this complex process. For example, *Streptococcus pneumoniae* produces negatively charged capsular polysaccharides that repel mucus to facilitate nasal colonization (*2*, *3*), while *Staphylococcus aureus* synthesizes uncharged poly-*N*-acetylglucosamine (PNAG) (*4*) or modifies its cell walls with *N*-acetylglucosamine (*5*, *6*) as adhesins to bind to nasal epithelial cells.

Among the most contagious nasal colonizers are pathogens belonging to the *Bordetella* species (bordetellae), including the human-specific pathogen *Bordetella pertussis* that causes pertussis (whooping cough) (*7*, *8*) and *Bordetella bronchiseptica* that causes respiratory infections in humans and many other animals (*9*, *10*). Despite high vaccine coverage in developed countries, *B. pertussis* still colonizes the host nasopharynx to sustain transmission (*11*–*14*). Similarly, *B. bronchiseptica* effectively colonizes and persists in the nasal cavities of mice (*15*, *16*), and *B. bronchiseptica* whole-cell vaccination fails to efficiently protect against nasal colonization (*17*).

Previous research on bordetellae has primarily focused on lower respiratory tract infection (*18*, *19*). These studies identified multiple bacterial proteins (often referred to as virulence factors), such as filamentous hemagglutinin (*20*–*22*), fimbriae (*23*), pertussis toxin (*24*), pertactin (*21*), tracheal colonization factor A (*25*), and adenylate cyclase toxin (*26*), all of which contributed, in varying degrees, toward the establishment of infections in the lower respiratory tract. Glycans such as O-antigen and Bordetella polysaccharide (Bps) polysaccharide have also been found to be involved in lower airway infection and in resisting complement-mediated killing (*27*, *28*) or in assisting biofilm formation (*29*, *30*). In contrast, much less is known about how bordetellae initially colonize the upper respiratory tract, a critical step in the transmission of these highly infectious pathogens.

**Fig. 1.**
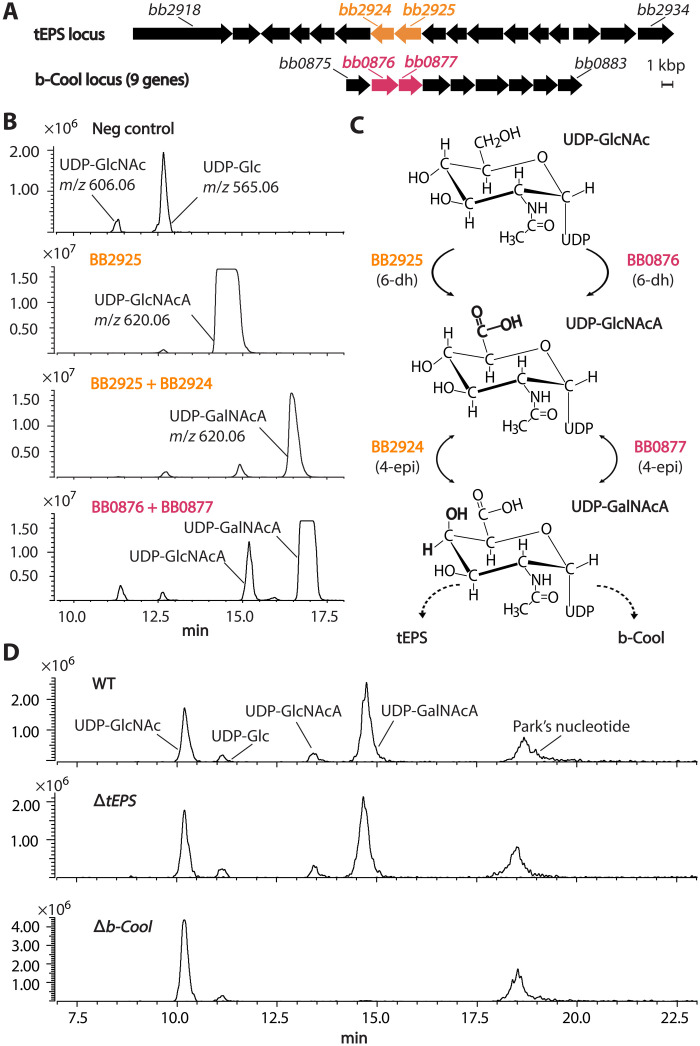
*bb2925*, *bb2924*, and their homologs *bb0876* and *bb0877* encode UDP-GlcNAc 6-dehydrogenase and UDP-GlcNAcA 4-epimerase, respectively, in *B. bronchiseptica*. Two genes involved in UDP-sugar biosynthesis in the tEPS locus were found to have homologs in another nine-gene locus that we named b-Cool (**A**). Enzymatic activities of BB2925, BB2925-BB2924 coexpression, and BB0876-BB0877 coexpression were examined (**B**). Both pairs of enzymes were shown to convert UDP-GlcNAc to UDP-GlcNAcA and subsequently to UDP-GalNAcA (**C**). LC-MS analyses of NDP-sugar extracted from the *Bb* wild type (WT) and two mutants deleted with either glycan locus (Δ*tEPS* and Δ*b-Cool*) showed that the wild type and Δ*tEPS* had identical NDP-sugar profiles, while Δ*b-Cool* did not produce any detectable UDP-GlcNAcA and UDP-GalNAcA (**D**). Note that slight differences in elution times due to different Hilic columns and times of acquisition. All experiments were independently performed three or more times.

In addition to the O-antigen and Bps polysaccharides, analyses of *Bordetella* genomes have predicted the presence of other glycans with unknown structures and functions (*31*, *32*). We have previously identified a glycan involved in transmission, named transmission-related extracellular polysaccharide (tEPS) (*33*). While studying the biosynthetic locus of tEPS, we determined the enzymatic function of two genes, which encode nucleotide sugar synthases forming uridine 5′-diphosphate–*N*-acetylglucosamine uronic acid (UDP-GlcNAcA) and UDP–*N*-acetylgalactosamine uronic acid (UDP-GalNAcA) (Fig. 1, A to C). The tEPS locus deletion mutant Δ*tEPS*, however, still produces those two nucleotide sugars (Fig. 1D). This can be explained by the presence of two homologs of nucleotide sugar synthases located in another nine-gene locus (Fig. 1, A to C). Here, we provide evidence that this previously uncharacterized nine-gene locus is responsible for producing a glycan involved in nasal colonization, which we name bordetellae colonization oligosaccharide (b-Cool). This is the first evidence of a glycan involved in early nasal colonization in bordetellae.

## RESULTS

### The *Bordetella* genome contains two glycan loci using similar UDP-sugar pathways

We have previously shown that a 17-gene locus, tEPS, contributes to the transmission of *B. bronchiseptica* (*33*). Two genes within tEPS, *bb2925* and *bb2924*, were predicted to function in nucleotide sugar biosynthesis. To investigate their enzymatic activities, we cloned and expressed *bb2925* and *bb2924* in *Escherichia coli*. The nucleotide sugars were extracted and analyzed by liquid chromatography tandem mass spectrometry (LC-MS/MS) (*34*). Compared to a negative control, a unique peak eluting from a Hilic column at 15 min with a mass/charge ratio (*m*/*z*) 620.0 was observed in *E. coli*–expressing BB2925 (Fig. 1B). MS/MS ions at *m/z* 305.0 and 402.9 are consistent with predicted fragmentation of a UDP-sugar into [UMP-H_2_O-H]^−^, [UDP-H], respectively (fig. S1B). Coexpression of BB2925 and BB2924 resulted in a substantially decreased level of the 15-min peak and accumulation of another *m/z* 620.0 peak at 17 min with identical MS/MS fragmentation (fig. S2B). Subsequent one-dimensional (1D) and 2D nuclear magnetic resonance (NMR) analyses of enzyme reaction products concluded that BB2925 is a 6-dehydrogenase converting UDP-GlcNAc and nicotinamide adenine dinucleotide (NAD^+^) to UDP-GlcNAcA (fig. S1) and reduced form of NAD^+^. BB2924 is a 4-epimerase and reversibly converts UDP-GlcNAcA to UDP-GalNAcA (fig. S2).

**Fig. 2.**
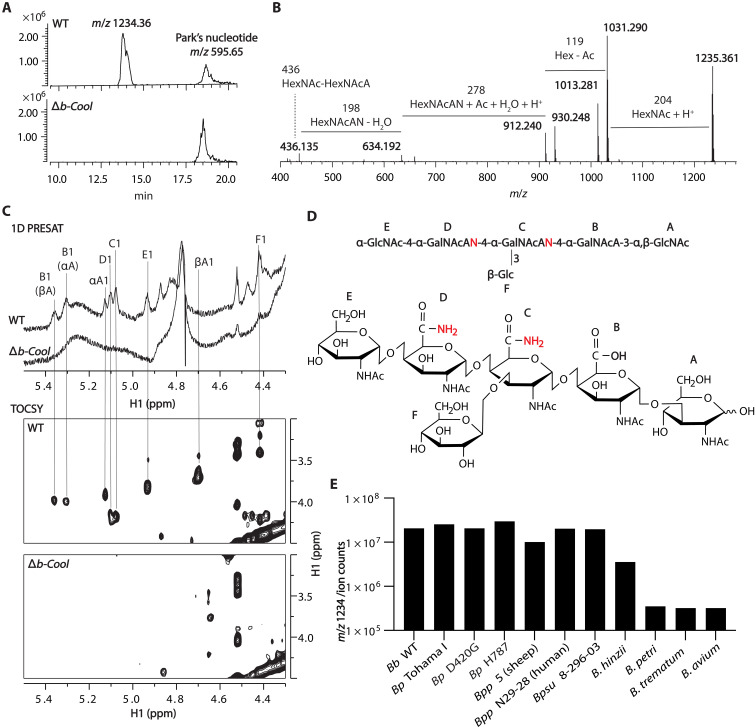
The b-Cool glycan (m/z 1234) is the product of the nine-gene locus. Soluble glycan extracts from the *B. bronchispetica* wild type or Δ*b-Cool* mutant were analyzed by LC-MS/MS using an Amide-Hilic column. A molecule with *m*/*z* 1234.36 was present in the wild type but absent from Δ*b-Cool* mutant (**A**). MS/MS fragmentation of *m*/*z* 1234.36 indicates a hexasaccharide (**B**). 1D PRESAT and 2D TOCSY high-resolution magic angle spinning (HR-MAS) NMR showed the presence of b-Cool in intact wild-type cells but not Δ*b-Cool* (**C**). The chemical structure of b-Cool was determined by 2D NMR (**D**). In addition, b-Cool was detected in cultures of a wide range of bordetellae strains (*Bb*, *B. bronchiseptica*; *Bp* Tohama I, *B. pertussis* lab strain; *Bp* D420G and H787, *B. pertussis* recent clinical isolates; *Bpp*, *B. parapertussis*; *Bpsu*, *B. pseudohinzii*) (**E**). All experiments were independently performed two or more times.

**Fig. 3.**
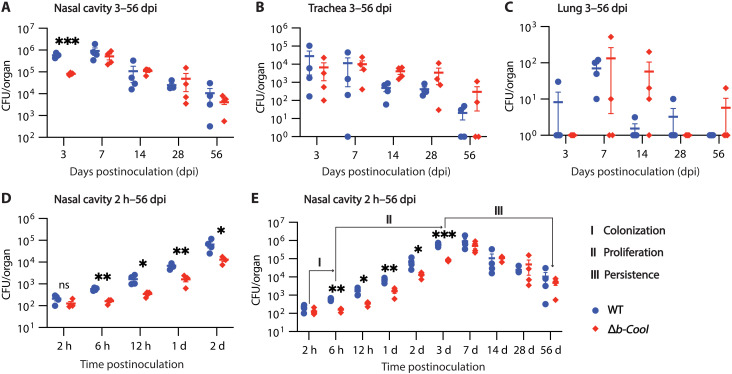
The nine-gene b-Cool locus is crucial for *Bordetella* early nasal colonization in mice. Around 500 CFU of the wild type or Δ*b-Cool* was delivered intranasally to C57BL/6 mice. Mice were euthanized periodically at 3, 7, 14, 28, and 56 dpi; the nasal cavity (**A**), trachea (**B**), and lung (**C**) were excised; and CFU was determined. A separate group of mice was inoculated similarly and euthanized at 2 hours, 6 hours, 12 hours, 1 dpi, and 2 dpi; and the nasal cavity CFU was examined (**D**). A combination of nasal cavity CFU from 2 hours to 56 dpi was shown as well (**E**). Data at each time point represent four biological replicates. The 3-dpi experiment was independently performed more than three times. The other experiments were independently performed two or three times. *P < 0.05, **P < 0.01, and ***P < 0.001.

Unexpectedly, the tEPS deletion mutant, Δ*tEPS*, showed a UDP-sugar profile similar to that of the wild type despite the deletion of the whole tEPS locus (Fig. 1D). After testing various growth conditions, Δ*tEPS* mutant consistently showed similar or only slightly reduced levels of UDP-GlcNAcA and UDP-GalNAcA when compared to the wild type (fig. S3). This suggested that alternative gene sources contribute to the synthesis of these NDP-sugars. Analyses of the *B. bronchiseptica* genome showed that *bb0876* and *bb0877* are homologs of *bb2925* and *bb2924*, respectively, sharing 99% amino acid sequence identity. These genes are located within a nine-gene locus that also encodes five putative glycosyltransferases (BB0875, BB0879, BB0881, BB0882, and BB0883), a putative asparagine synthase (BB0880), and a putative membrane protein (BB0878) (Fig. 1A). The expression of BB0876 and BB0877 in *E. coli* also led to production of UDP-GlcNAcA and UDP-GalNAcA (Fig. 1B). To determine if *bb0876* and *bb0877* are the alternative sources of UDP-GlcNAcA and UDP-GalNAcA, the nine-gene locus was deleted by allele exchange (fig. S4). The deletion of this locus resulted in the complete absence of UDP-GlcNAcA and UDP-GalNAcA, indicating that under laboratory growth conditions, the production of these NDP-sugars in *B. bronchiseptica* is primarily controlled by this nine-gene locus (Fig. 1C and fig. S3). We next investigated the structure and biological function of the glycan produced by the nine-gene locus. On the basis of its structure and function (explored below), we named the nine-gene locus b-Cool (Figs. 2 and 3).

### The b-Cool locus produces an oligosaccharide conserved across bordetellae

To characterize the glycan product of the b-Cool locus, we cultured *B. bronchiseptica* wild type and the b-Cool locus deletion mutant Δ*b-Cool*, extracted glycans and metabolites from cells, and analyzed the extracts by LC-MS/MS. An abundant peak with a *m*/*z* 1234.4, eluting from the Hilic column at 14 min, was detected in the wild type but absent from Δ*b-Cool* (Fig. 2A). MS/MS fragmentation indicated the peak is a glycan likely composed of six sugars: one hexose, two *N*-acetylhexosamine, and initially suspected as three HexNAcA (*N*-acetylhexosamino uronic acid) residues (Fig. 2B). However, the calculated mass of the six sugars suggested the presence of two CO-NH_2_ (uronate-amide) instead of COOH (uronic acid) groups in the putative HexNAcA residues.

The *m*/*z* 1234.4 glycan was purified from *B. bronchiseptica* wild type by charge and size chromatographies and then subjected to 1D and 2D NMR analyses. NMR data suggested that the *m*/*z* 1234.4 glycan structure is GlcNAc(α1-4)GalNAcAN(α1-4) [Glc(β1-3)]GalNAcAN(α1-4)GalNAcA(α1-3)GlcNAc(α/β) (Fig. 2D, table S1, and fig. S5). In addition, the *m*/*z* 1234.4 glycan was also detected in the intact wild-type bacteria, but not Δ*b-Cool*, by high-resolution magic angle spinning NMR (HR-MAS NMR) (Fig. 2C). The glycan’s anomeric protons (H1) and H1-H3 correlations were resolved by 1D HR-MAS NMR and 2D TOCSY HR-MAS NMR in the wild type while absent in Δ*b-Cool* (Fig. 2C and fig. S6). Moreover, the *m*/*z* 1234.4 glycan was also detected in two other glycan mutants, Δ*tEPS* and O-antigen deletion mutant Δ*wbm* by HR-MAS NMR (fig. S6), indicating that b-cool oligosaccharide production is independent of the tEPS and O-antigen pathways.

The b-Cool locus is conserved across *Bordetella* species, including the human pathogen *B. pertussis*. To determine if the unique glycan is produced in other *Bordetella* species, we cultured multiple bordetellae, including *B. pertussis* (laboratory strains and 11 recent *B. pertussis* clinical isolates), *Bordetella parapertussis*, *Bordetella pseudohinzii*, *Bordetella trematum*, *Bordetella petrii*, *Bordetella hinizii*, and *Bordetella avium*. After 24 hours of growth, glycans were isolated and analyzed by LC-MS/MS. Under laboratory growth conditions, all cultured bordetellae, including 11 *B. pertussis* clinical isolates, produced a *m*/*z* 1234.4 glycan (Fig. 2E and fig. S7) with the same elution time from the Hilic column. Furthermore, the *m*/*z* 1234.4 glycans from different bordetellae have similar MS/MS patterns as the one isolated from the *B. bronchiseptica* wild-type strain. These results indicate that b-Cool (*m*/*z* 1234.4) is conserved across disparate bordetellae, suggesting its critical function in all *Bordetella* spp.

### B-Cool is crucial for early nasal colonization

To investigate the biological function of the b-Cool glycan, we used an established mouse model that enables the study of natural infection from low-dose inoculum (*33*). C57BL/6 mice were intranasally inoculated with either *B. bronchiseptica* wild-type or Δ*b-Cool* mutant. Bacteria were recovered from respiratory organs (nasal cavity, trachea, and lung) at different times postinoculation. Bacterial colony-forming units (CFUs) were enumerated by plating on Bordet–Gengou (BG) agar. At 3 days postinoculation (dpi), there were about 10-fold fewer CFU, i.e., 1 log_10_ decrease, of Δ*b-Cool* than the wild type in the nasal cavity, but similar numbers of the wild type and mutant were found in the trachea and lungs (Fig. 3, A to C). No significant differences between the wild type and the mutant were observed at later time points in any organ, indicating a role of b-Cool in the early steps (before day 3) of nasal colonization.

To better define the role of b-Cool during these early stages of colonization, we compared the nasal cavity CFU of the wild type and Δ*b-Cool* at multiple earlier time points from 2 hours to 2 dpi. At 2 hours postinoculation, similar numbers of wild type and Δ*b-Cool* were recovered, confirming the consistency of our inoculation. However, by 6 hours postinoculation, the wild-type CFU had tripled, but the mutant did not increase in numbers. At the following four consecutive time points from 12 hours to 3 dpi, both strains increased in numbers, but we consistently observed 75 to 85% fewer CFU of the Δ*b-Cool* than the wild type (Fig. 3, D and E), contributing to the rigor of the observation. After the initial delay in colonization from 2 to 6 hours, the CFU levels of Δ*b-Cool* appeared to increase at a similar rate as the wild type, thereafter suggesting a key role of b-Cool in the critical early period within 6 hours of bacteria-host interaction. It appears that b-Cool is the only glycan critical for the early colonization because we do not observe a similar defect in the tEPS deletion mutant Δ*tEPS* or O-antigen deletion mutant Δ*wbm* (fig. S8). In the context of this study, we defined 2 to 6 hours postinoculation as the colonization stage, 6 hours to 3 dpi as the proliferation stage, and beyond 3 dpi as the persistence stage (Fig. 3E).

#### *The nasal colonization defect of* Δ*b-Cool is not due to disruption of NDP-sugar flux or other glycan pathways*

The deletion of the nine-gene b-Cool locus led to disruption of normal NDP-sugar flux, resulting in the absence of UDP-GlcNAcA (*m*/*z* 620.06) and UDP-GalNAcA (*m*/*z* 620.06) (Fig. 1D and fig. S9B) and the absence of UDP-HexA-2,3NAc with *m*/*z* 661.06 (fig. S9B). Alteration in the NDP-sugar flux within the Δ*b-Cool* mutant may affect other glycan pathways. In addition, the first gene of the b-Cool operon, *bb0875*, encodes an apparent phosphate-sugar transferase. Its product, C55-PP-GlcNAc, might be shared with other pathways (fig. S9A). To determine if the Δ*b-Cool* defect is due to alteration in NDP-sugar flux or other glycan pathways, we generated three complementation genetic constructs. One construct contains the two NDP-sugar synthases (*bb0876* and *bb0877*), the other contains the first five genes of the b-Cool operon, including *bb0875* and the two NDP-sugars synthases, and the last one contains the complete nine-gene b-Cool locus. All three complementation constructs restore the UDP-HexNAcA (*m*/*z* 620.05) and UDP-HexNAcA-2,3NAc (*m*/*z* 661.09), while only the nine-gene complementation restores the production of b-Cool (*m*/*z* 1234.4) (fig. S9, B and C). Subsequently, we compared the nasal colonization of the wild type, Δ*b-Cool*, and complemented Δ*b-Cool* at 3 dpi. Both the two-gene and five-gene complemented Δ*b-Cool* strains show similar colonization defects to the uncomplemented Δ*b-Cool* mutant, and the nine-gene complemented Δ*b-Cool* showed significantly increased nasal colonization compared to uncomplemented or partially complemented Δ*b-Cool* (fig. S9, D and E). These results indicate that the nasal colonization defect of Δ*b-Cool* is not due to disruption of NDP-sugar flux or other glycan pathways but likely due to loss of the b-Cool glycan.

### B-Cool is involved in the colonization of nasal but not tracheobronchial epithelia in the ALI

**Fig. 4.**
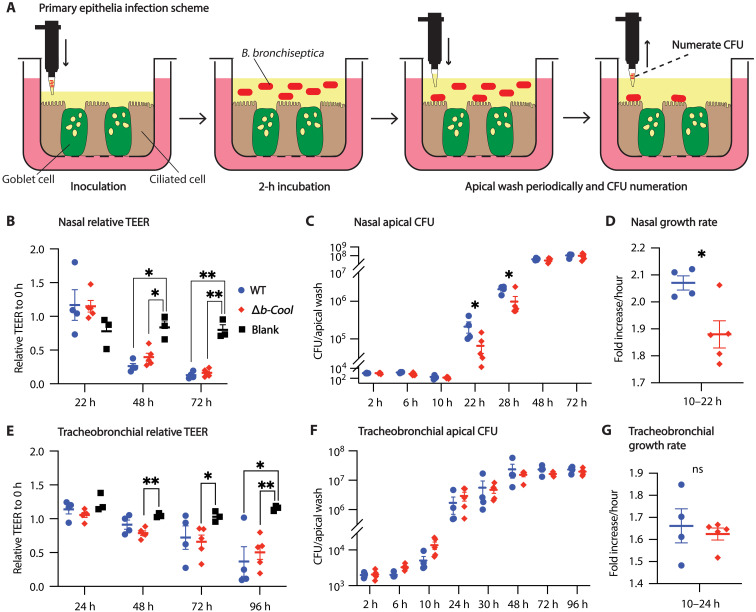
Δ*b-Cool* is impaired in early growth in primary nasal, but not tracheal, epithelia cultured in ALI. *Bb* wild type or the Δ*b-Cool* mutant was inoculated to primary mouse nasal or tracheobronchial epithelial cultured in an ALI. In the cartoon, the green colored cells represent goblet cells, brown colored cells represent ciliated cells, and red rounded rectangles represent *Bb* (**A**). TEER was monitored as an indicator of tissue integrity. Both the wild type and Δ*b-Cool* mutant compromise the TEER of nasal (**B**) or tracheobronchial (**E**) epithelia. The Δ*b-Cool* mutant exhibited a colonization defect relative to the wild type when inoculated to the apical of nasal epithelia (**C** and **D**) but showed no defect on the apical of tracheal epithelia (**F** and **G**). Data at each time point represent four biological replicates. All experiments were independently performed three times. *P < 0.05 and **P < 0.01. ns, not significant.

The nasal and tracheobronchial environments differ in various aspects, including nonbiological factors such as temperature and humidity (*35*) and biological factors such as different proportions of distinct epithelial cell types (*36*) and varying levels of Muc5Ac (*37*). We hypothesized that the observed colonization defect of Δ*b-Cool* in the nasal cavity but not in the trachea (Fig. 3, A and B) was related to the distinct epithelial cells and their functions in these two niches rather than nonbiological variables (humidity, temperature, etc.). To simplify the complexity of in vivo systems and to have precise control of nonbiological variables, we used primary mouse epithelia, which were isolated from nasal and tracheobronchial tissue and subsequentially cultured and differentiated in an air-liquid interface (ALI). While it is customary to use primary mouse tracheal or tracheobronchial epithelia ALI culture (*38*, *39*), we have also tackled technical hurdles to develop and establish primary mouse nasal epithelia ALI culture. We harvested, grew, and differentiated mouse nasal and tracheobronchial epithelia in an ALI as described (*38*, *40*) with modifications (see Materials and Methods for details). We then determined the impact of *Bordetella* on tissue barrier function by monitoring the trans-epithelial electrical resistance (TEER). Infection with either the wild-type or Δ*b-Cool* mutant led to significantly reduced TEER of nasal or tracheobronchial epithelia by 48 to 72 hours postinoculation (Fig. 4, B and E, and fig. S10). In the tracheobronchial epithelia, we observed no significant difference in colonization between *B. bronchiseptica* wild type and Δ*b-Cool* (Fig. 4, F and G). In nasal epithelia, we observed no difference in apical CFU of wild type and Δ*b-Cool* between 2 and 10 hours, when bacterial CFU are relatively low, but we observed a significant defect of Δ*b-Cool* at 22 and 28 hours postinoculation (Fig. 4, C and D), a timeframe that corresponds to the early defect in mouse nose (Fig. 3). We did not observe a difference in the ability of wild type and Δ*b-Cool* to bind to mouse nasal epithelia (fig. S11). The observation of a colonization defect of Δ*b-Cool* in nasal epithelia but not in tracheobronchial epithelia agrees with the colonization studies in mice (Fig. 3) and also provides an experimental system to further define the mechanistic basis for the role of b-Cool in early colonization.

### B-Cool promotes nasal colonization in the presence of airway mucins

**Fig. 5.**
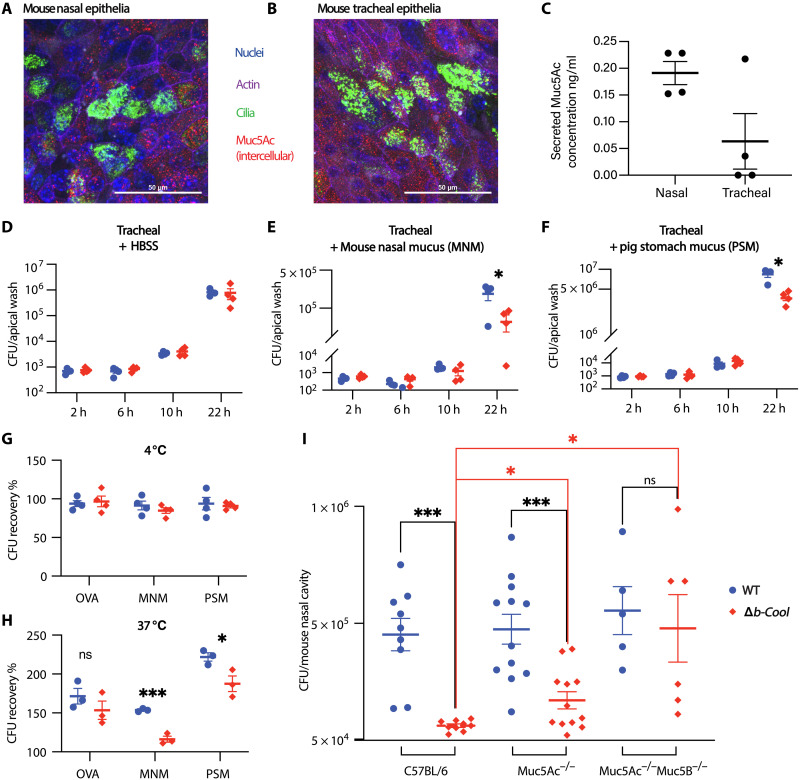
B-Cool promotes colonization in presence of airway mucins. Although mouse nasal epithelia (MNE) (**A**) and mouse tracheobronchial epithelia (MTE) (**B**) shared similar morphology, MNE secrete more mucus than MTE to the apical surface, as quantified by Muc5Ac ELISA (**C**). To further evaluate the role of mucus in *B. bronchiseptica* colonization, identical CFUs of wild type or Δ*b-Cool* were inoculated to MTE, and either control HBSS or mucus was supplemented before inoculation and after each apical wash. Δ*b-Cool* showed no colonization defect when supplemented with HBSS (**D**) but showed a profound colonization defect when supplemented with MNM (**E**) and PSM (**F**). To further investigate the interaction of wild type or Δ*b-Cool* with mucus, ovalbumin (OVA), MNE, or PSM was coated to 96-well plates, and identical CFUs of wild type or Δ*b-Cool* were inoculated to corresponding wells. Plates were incubated at 4° and 37°C for 1 hour, and unbound bacteria were recovered and plated for CFUs. Identical amounts of wild type and Δ*b-Cool* were recovered from each group at 4°C (**G**). At 37°C, similar amounts of wild type and Δ*b-Cool* were recovered from OVA-coated wells, while significantly less Δ*b-Cool* was recovered than the wild type from mucus-coated wells (**H**). Last, wild type or Δ*b-Cool* was inoculated to C57BL/6 mice (wild-type mice) or mucin-deficient mice (Muc5Ac^−/−^ and Muc5Ac^−/−^Muc5B^−/−^) and bacterial CFUs in the nasal cavity were examined at 3 dpi. The colonization defect of Δ*b-Cool* was partially rescued in Muc5Ac^−/−^ mice and nearly completely rescued in the mucins double deletion mice, Muc5Ac^−/−^Muc5B^−/−^ (**I**), indicating that b-Cool provide resistance to both mucins (Muc5Ac and Muc5B). Data at each time point represent four or more biological replicates. All experiments were independently performed two or three times. *P < 0.05 and ***P < 0.001.

Because of the observation of a colonization defect of Δ*b-Cool* in the nasal but not tracheobronchial epithelia, we hypothesized that a nasal-specific host factor was involved. We first compared the morphology of mouse primary nasal and tracheobronchial epithelia by immunostaining and confocal microscopy (Fig. 5, A and B). While we did not observe a difference in their morphology, we observed a gel-like material rapidly accumulating on the apical surface of mouse nasal epithelia but not tracheobronchial epithelia. The gel-like material was determined by enzyme-linked immunosorbent assay (ELISA) to contain Muc5Ac and, hence, was likely mucus. Subsequently, Muc5Ac levels in the apical wash of both mouse nasal and tracheobronchial epithelia were quantified by ELISA, and the data suggest that mouse nasal epithelia secrete more Muc5Ac than tracheobronchial epithelia (Fig. 5C). To test if the mucus is the host factor that leads to the colonization defect of Δ*b-Cool*, we supplemented external mucus to tracheobronchial epithelia before inoculation and post each apical wash. Similarly to what we observed in mouse nasal epithelia (Fig. 4C), we also observed a colonization defect when tracheobronchial epithelia were supplemented with mouse nasal mucus (MNM) and pig stomach mucus (PSM), but not in the control, which is supplemented with Hank’s balanced salt solution (HBSS) (Fig. 5, D to F). We also observed a significant colonization defect of Δ*b-Cool* in normal human bronchial epithelial (NHBE) culture in ALI when mucus was present (fig. S12). These data further suggest that mucus contributes to the colonization defect of Δ*b-Cool*.

To further evaluate the interaction of wild type and Δ*b-Cool* with mucus, we established a simplified assay by coating 96-well plates with mucus or ovalbumin (control). Identical amounts of wild type and Δ*b-Cool* were inoculated into the coated plates, and after incubation at either 37° or 4°C for 1 hour, the bacteria were recovered for CFU enumeration. We recovered similar amount of wild type and Δ*b-Cool* (above 75% compared to the inoculum) from each group at 4°C, suggesting that Δ*b-Cool* has a similar ability as the wild type to repel mucus at 4°C. We recovered significantly less Δ*b-Cool* than wild type in mucus-coated wells, but not in ovalbumin-coated wells, at 37°C (Fig. 5, G and H). These findings confirm that Δ*b-Cool* exhibits a colonization defect in the presence of mucus.

Airways mucus consists of two main mucin glycoproteins, Muc5Ac and Muc5B. Both MNM and PSM are enriched in Muc5Ac, indicating the possible role of Muc5Ac in resisting the efficient colonization of Δ*b-Cool*. To test this hypothesis, we first compared the nasal colonization of wild type and Δ*b-Cool* in wild-type mice (C57BL/6) and Muc5Ac-deficient mice (Muc5Ac^−/−^). However, only minor rescue (Fig. 5I) of the Δ*b-Cool*’s defect was observed. Because Muc5B is up-regulated in Muc5Ac^−/−^ mice to compensate the loss of Muc5Ac (*41*), we compared the colonization in mice deficient in both mucins (Muc5Ac^−/−^Muc5B^−/−^). We observed a near complete rescue of the defect of Δ*b-Cool* in the double mucin-deficient mice (Fig. 5I). Together, these data suggest that b-Cool may function in resisting airway mucins.

### B-Cool is critical for transmission

**Fig. 6.**
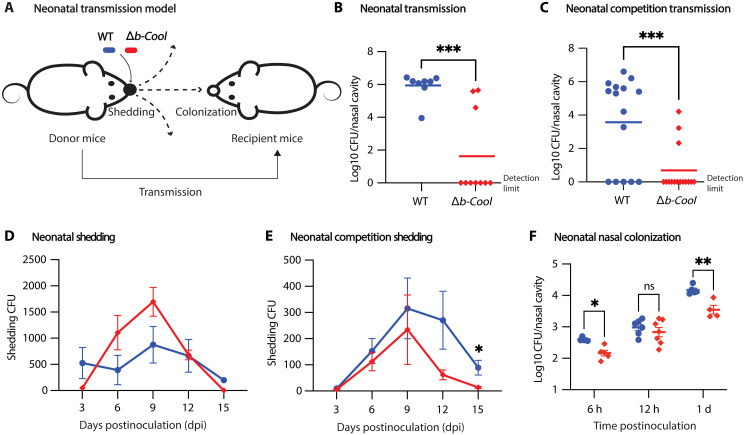
The b-Cool locus is critical for transmission and nasal colonization. A neonatal transmission model was developed with part of the newborn mice inoculated (donors) and the rest uninoculated (recipients) (**A**). Half of the 5-day-old newborn mice were inoculated with *Bb* wild type or Δ*b-Cool*, and the colony was maintained for 21 days before sacrifice (**B**). Alternatively, two to four donors per cage were inoculated with a mixture of *Bb* wild type and Δ*b-Cool* at a 1:1 ratio. The colony was maintained for 16 days before weaning and sacrificed at 21 dpi (**C**). Bacterial shedding was assessed during the transmission experiment (**D**) or competition transmission experiment (**E**). Five-day-old mice were inoculated with *Bb* wild type or Δ*b-Cool* and euthanized periodically to assess neonatal nasal colonization (**F**). The bacteria CFU in the respiratory organs of the donor mice in the co-inoculation transmission experiments are shown in fig. S13. Data at each time point represent four or more biological replicates. All experiments were independently performed twice. *P < 0.05, **P < 0.01, and ***P < 0.001.

We anticipated that the Δ*b-Cool* mutant would have impaired transmission due to its deficient nasal colonization. To determine the comparative transmission abilities of the wild type versus Δ*b-Cool*, we used a neonatal mouse transmission model (*42*) with modifications (Fig. 6A). The wild-type strain transmitted from challenged mice to 100% of cohoused (exposed) mice, but the Δ*b-Cool* transmitted to only 30% of exposed mice (Fig. 6B). To both confirm this defect and to explore whether b-Cool might function in trans, a competition assay was conducted. The wild-type and the Δ*b-Cool* mutant were co-inoculated at a 1:1 ratio into donor mice. The wild-type strain was transmitted to 10 of the 15 exposed mice (67%), but the Δ*b-Cool* strain was transmitted to only 3 of the 15 (20%) (Fig. 6C).

To better understand the nature of Δ*b-Cool*’s defect in transmission, we examined two aspects of transmission: shedding (bacteria recovered from the outer surface of the nose) and nasal colonization. We observed similar bacterial shedding of the wild type and Δ*b-Cool* at all time points, with the peak of shedding of both at 9 dpi (Fig. 6D), suggesting that b-Cool is not essential for bacterial shedding. Mirroring what was observed in adult mice, we also observed a similar nasal colonization defect of Δ*b-Cool* in the neonatal mice at 6 hours post-inoculation and 1 dpi (Fig. 6F). These data indicates that the transmission defect of Δ*b-Cool* observed here is more likely a consequence of a defect in nasal colonization than in shedding.

Last, we investigated the relative contribution of b-Cool and tEPS to transmission. We have previously shown that Δ*tEPS* had a transmission defect due to impaired bacterial shedding (*33*). We compared the transmission of WT, Δ*b-Cool*, and Δ*tEPS* side-by-side by using an alternative model (fig. S14). The wild-type strain transmitted to 100% of exposed mice, while Δ*b-Cool* transmitted to 30% of exposed mice, consistent with observations in the neonatal transmission model. The Δ*tEPS*, however, transmitted to 58% of exposed mice, which is higher than previously reported (*33*). This variation in transmission could be influenced by several variables, including humidity, the age of mice, and resident nasal microbiota. Hence, the relative transmission defects among different mutants can only be accurately compared within the same batch of mice. Together, our data suggest that b-Cool and tEPS contribute to distinct aspects of transmission, specifically nasal colonization and shedding, respectively. Notably, b-Cool appears to contribute more substantially to facilitating efficient transmission than tEPS.

## DISCUSSION

Here, we purified and determined the chemical structure of a novel glycan and revealed its role in early nasal colonization. Furthermore, we showed that b-Cool glycan is produced in various *Bordetella* species, including clinical isolates of *B. pertussis* (Fig. 2E and fig. S7). The high nucleotide sequence identity of the nine-gene b-Cool locus across the *Bordetella* species and their high production of the energetically costly b-Cool glycan suggest that these genes have critical functions across bordetellae. *B. bronchiseptica* and *B. pertussis* are closely related subspecies that colonize the respiratory tract and cause disease in humans (*43*). Although *B. pertussis*, which is specialized to infect humans, has lost nearly 25% of its genome in the process (*31*), the *b-Cool* locus is exquisitely preserved since their divergence, indicating that b-Cool is essential and strongly suggesting that it serves a conserved role.

In *Bordetella* spp., two pairs of genes are involved in the biosynthesis of UDP-GlcNAcA and UDP-GalNAcA: one pair of genes (*bb0876*-*bb0877*) is located in the b-Cool locus and a highly similar pair in the tEPS locus (*bb2925*-*bb2924*) (Fig. 1). A prior study suggested that the homologs of BB2925 and BB0876 in *B. pertussis*, BP3150 and BP1629, are 6-dehydrogenases that are capable of using UDP-Glc, UDP-GlcNAc, and UDP-GalNAc as substrates in vitro (*44*). However, our NMR analyses show that the enzymatic product of BB2925 and BB0876 is UDP-GlcNAcA. The presence of two identical UDP-GlcNAcA and UDP-GalNAcA biosynthesis pathways suggested that b-Cool and tEPS evolved separately to fulfill different functions.

Strikingly, Δ*b-Cool* showed a substantial delay in early nasal colonization as early as 6 hours postinoculation, which led to significant nasal colonization defects from 6 hours to 3 dpi (Fig. 3E). This early nasal colonization defect underscores the role of b-Cool glycan as acting very early during infection. Furthermore, we observed a 10-fold reduction in bacterial burden of the Δ*b-Cool* compared to that of the wild type at the early stages. This represents a notable biological difference in the context of *B. bronchiseptica* nasal colonization. *B. bronchiseptica* is known to be an efficient and persistent colonizer in mice, and even the whole cell vaccination could only reduce nasal colonization by 10- to 100-fold despite conferring near complete protection in the lower airways (*17*).

In addition, Δ*b-Cool* demonstrated a profound 70% reduction in transmission (Fig. 6, B and C), suggesting that disrupting the b-Cool pathway not only hinders an early step in nasal colonization but also substantially decrease the likelihood of transmission events. These findings highlight the contribution of early-stage colonization to transmission, which agrees with prior reports that the early stage of pertussis transmission is often asymptomatic but allows for efficient transmission (*13*, *45*).

The colonization defect of Δ*b-Cool* was observed exclusively in the nasal cavity and not in the trachea despite the proximity of these two organs. When controlling for nonbiological factors (such as temperature and humidity) ex vivo, we still observed colonization defects specific to nasal epithelial infection, while tracheobronchial epithelial infection remained unaffected. These findings validate that our novel primary epithelial systems in an ALI provide useful experimental models to quantify and study detailed and mechanistic aspects of host-pathogen interaction.

Our data show that the mouse nasal epithelia secrete more mucus than tracheobronchial epithelia, consistent with observation in human epithelial culture (*37*). However, it is somewhat unexpected to observe that Δ*b-Cool* was impaired in the presence of both PSM (Fig. 5, F and H) and NHBE mucus (figs. S11 and S12), suggesting that the inhibition effect of mucus on the Δ*b-Cool* strain is not specific to its type or origin.

Some bacteria produce negatively charged glycans to repel mucus and facilitate colonization (*1*–*3*). However, we found that similar amount of the wild type and Δ*b-Cool* (75 to 100%) could be recovered postincubation with mucus at 4°C (Fig. 5G). This suggests that Δ*b-Cool* is neither impaired in repelling mucus nor sensitive to mucus-mediated bacterial lysis. In addition, we did not observe Δ*b-Cool* being deficient in binding to nasal epithelia (fig. S11). While both the wild type and Δ*b-Cool* increased in CFU when incubated with mucus at 37°C, the wild type had a significantly greater increase than the mutant (Fig. 5H). Collectively, our results suggest that b-Cool promotes colonization in the presence of mucus, and it appears that airway gel-forming mucins (Muc5Ac and Muc5B) are the major factors involved (Fig. 5I).

While there are some structural differences between the nasopharynx of rodents and humans, both airways consist of pseudostratified ciliated columnar epithelium and both use a common defense mechanism known as the mucociliary escalator that captures and removes airway pathogens (*46*). In addition, we observed a colonization defect of the *B. bronchiseptica* Δ*b-Cool* mutant in NHBE (fig. S12), which actively secrete mucus. While currently the *B. pertussis* b-Cool mutants are not available, future studies investigating b-Cool mutants in *B. pertussis* using human nasal epithelial cultures will provide a clearer understanding of their important and conserved functions.

In summary, we have identified a unique hexasaccharide glycan, b-Cool, produced by diverse *Bordetella* species, including clinical isolates. We described the critical role of b-Cool glycan in the very early stages of infection, occurring within just a few hours after bacteria are introduced to their hosts. Our findings highlight the importance of early nasal colonization in the infection process and its role in transmission. Moreover, we demonstrated that host mucins inhibit bacterial colonization and the production of b-Cool counteracts this inhibition. These findings suggest that developing strategies to disrupt glycan functions, such as small-molecule inhibitors targeting b-Cool biosynthesis, could help address the urgent challenges posed by the highly transmissible *Bordetella* species.

## MATERIALS AND METHODS

### Culture of *Bordetella* species

Strains from different *Bordetella* species used in this study were stored in modified Stainer-Scholte (SS) medium (*47*) containing 16% glycerol at −80°C. Strains were routinely streaked on Bordet-Gengou (BG; Difco) agar containing 10% sheep blood (HemoStat) and incubated at 37°C for 36 to 48 hours. Hemolytic colonies were picked, inoculated in modified SS or LB (Difco) media, and agitated at 200 to 220 rpm at 37°C for the appropriate time.

### Genomic deletion and complementation of the b-Cool locus

*B. bronchiseptica* wild type (strain RB50) and mating strain *E. coli* SM10 (λpair) transformed with the deletion construct pEXΔ*b-Cool* were cultured in LB broth overnight, washed by phosphate-buffered saline (PBS; pH 7.4) three times, and then resuspended in 300 μl of PBS. The suspension of two strains was mixed on an agar plate (BG supplemented with 10 mM MgCl_2_) and incubated at 37°C for 6 to 8 hours. After incubation, bacteria were washed from the plate by PBS, serially diluted, and plated on BG agar with kanamycin (50 μg/ml) and incubated at 37°C for 36 to 48 hours. For analyses, 30 to 40 single hemolytic colonies were picked and streaked on BG agar with kanamycin (50 μg/ml). About 10 to 20 kanamycin resistance hemolytic colonies were screened by polymerase chain reaction (PCR) to isolate single crossover events. Two to three single crossover co-integrant colonies were streaked on sucrose counter-selection agar [LB agar with no NaCl, supplemented with 15% sucrose and kanamycin (50 μg/ml)] to induce a second single crossover event. Colonies (10 to 20) were selected and screened by PCR to identify and select deletion mutants. These were streaked on BG agar with kanamycin (50 μg/ml) to verify the hemolytic status and stored in 16% glycerol at −80°C.

*B. bronchiseptica* wild type or Δ*b-Cool* were transformed with complementation plasmids (table S2) by electroporation under the selection of gentamycin (20 μg/ml). A few single colonies were selected for analysis.

### Molecular cloning

Polymerase incomplete primer extension (PIPE) was used to clone all the plasmids in this study except for the nine-gene complementation construct (table S2) as described previously (*34*). Briefly, the parental plasmids and the gene of interest to be cloned were amplified by PCR using Q5 DNA polymerase [New England Biolabs (NEB)] with supplemented reagents. GC enhancer was added if necessary to help with amplification of GC-rich products. The PCR products (a mixture of parent vector and gene of interest) were treated with 1 μl of Dpn I (NEB) and incubated at 37°C for 45 min. Clones were transformed to chemical competent to *E. coli* DH10b cells. After heat shock (42°C for 30 s), *E. coli* was recovered (37°C 2 hours in LB) and plated on LB agar supplemented with corresponding antibiotics (table S2). Clones were selected, and the DNA sequence was verified.

The nine-gene complementation construct was synthesized by Synbio Technology. Briefly, a 5.6-kbps DNA fragments correspond to the sequence of *bb0880* to *bb0883* were synthesized and assembled into pBBR-pfhaB-*bb0875*-*bb0879*. The complete sequence of the nine-gene complementation construct was verified by sequencing.

### LC-MS/MS analysis of UDP-sugar and other metabolites

Metabolites were extracted from bacteria according to the previously published protocol (*34*). Briefly, 5 ml cell culture samples were centrifuged at 10,000*g*, 4°C, 5 min. Following medium removal, 1 ml of cold 2:2:1 solvent (methanol:acetonitrile:water mixture, 2:2:1 v/v) was added, and samples were kept at −20°C. After 1 hour, samples were vortexed and centrifuged at 14,000 rpm, 25°C, 5 min. The supernatant was dried by air flow at room temperature in a hood and redissolved in 100 μl of water. Samples were injected into an Accucore-Amide-HILIC column (Thermo Fisher Scientific, 4.6 mm by 150 mm). Chromatography and analysis were carried out as described (*34*) using a Shimadzu LC-MS–ion trap–time-of-flight. UDP-sugar standards at various concentrations were used to determine the limit of detection.

### Purification of UDP-GlcNAcA, UDP-GalNAcA, and b-Cool glycan

UDP-GlcNAcA and UDP-GalNAcA were purified from *E. coli* BL21 (DE3) harboring corresponding plasmids (pET28b-pT7-*bb2925* or pCDF-paraB-*bb2925*-*bb2924*). *E. coli* strains (100 ml of LB) were cultured [optical density at 600 nm (OD_600nm_) of 0.6 to 0.8], and gene expressions were induced by the addition of 0.5 mM isopropyl-β-D-thiogalactopyranoside or 6.6 mM l-arabinose at 37°C for 4 hours. Cultures were centrifuged (at 10,000*g*, 4°C for 5 min). The medium was discarded, and cell pellets were extracted with freezer-cold 10-ml reagent (methanol:acetonitrile:water, 2:2:1), processed, and dried as above. UDP-GlcNAcA and UDP-GalNAcA were separated by a Q15 anionic exchange column as described previously (*48*), and peaks were collected and then lyophilized twice and reconstituted in 550 μl of D_2_O for NMR analyses.

B-Cool glycan was purified from 1 liter of *B. bronchiseptica* wild-type culture grown in LB at 37°C for 24 hours. *B. bronchiseptica* wild-type cells were similarly extracted by the 2:2:1 solvent, dried, and dissolved in 3 ml of water. To purify b-Cool, samples (6 × 500 μl) were injected into a Q15 anion exchange column (5 × 50 cm), and the flow through from 1 to 3 min was collected, lyophilized, and dissolved in 150 μl of water. The Q15 flowthroughs (6 × 100 μl) were injected at 0.5 ml/min into a P2 gel filtration column (Bio-Rad, packed in 4.6 mm by 250 mm) eluted with 20 mM ammonium formate. The P2 column fractions were analyzed by LC-MS, and b-Cool–containing fractions were combined, lyophilized, and reconstituted in 40 μl of D_2_O for NMR analyses.

### 1D and 2D NMR

For NMR analyses, samples were reconstituted in D_2_O. A 10 μM deuterated sodium trimethylsilyl propane sulfonate was spiked into samples as chemical shift references (δH 0.00 parts per million). For N15 natural abundance experiments, purified b-Cool was reconstituted in 85.5%H_2_O/9.5%D_2_O/5% deuterated acetic acid. Acetic acid was added to minimize amide proton exchange. All experiments were conducted in Bruker 600 or 800 MHz instruments at 25°C unless otherwise specified.

For HR-MAS NMR, *B. bronchiseptica* wild type or mutants were cultured in 10 ml of LB at 37°C for 24 hours and centrifuged (at 10,000*g*, 4°C for 5 min), and the medium was discarded. The cell pellets were washed twice (PBS made in D_2_O) and resuspended with 10 to 20 μl of PBS in D_2_O. The viscous cell pellets were transferred and packed into a 4-mm rotor. The spectra were acquired on a Bruker 600 MHz instrument at 25°C with a CMP-HRMAS probe (magic angle spinning at 5000 Hz). The spectra were processed with Mnova (MestreLab Research).

### Mouse colonization and transmission models

The mouse colonization and transmission models were routinely used in previous publications (*33*, *49*, *50*). All animal experiments were conducted strictly following the animal usage protocols (approved by UGA Institutional Animal Care and Use Committee, reference numbers A2022 04-001-Y3-A5 and A2022 04-022-Y3-A7). *B. bronchiseptica* wild type, Δ*b-Cool*, Δ*b-Cool* complementation strains, Δ*tEPS*, or Δ*wbm* were grown in SS medium to OD_600nm_ of 0.5 to 1.0. The OD_600nm_ of each strain was diluted to 0.1 by PBS, which was equivalent to 10^5^ CFU/μl based on agar plating. The culture with 10^5^ CFU/μl bacteria was further serial diluted to 100 CFU/μl, and 5 μl (100 CFU/μl) of bacteria culture was administrated intranasally to mice. The exact inoculum CFUs were determined by plating on BG agars.

For the colonization model, adult C57BL/6 mice (4 to 6 weeks old, the Jackson Laboratory, male or female) were inoculated with 5 μl (100 CFU/μl) of bacteria culture and euthanized at different times postinoculation. Three airway organs (nasal cavity, trachea, and lung) of each mouse were excised and placed in 1 ml of cold PBS. Tissues were further cooled in ice and then homogenized by a Bead Mills homogenizer (4.5 m/s, 45 s). Samples were plated on BG agar containing 10% sheep blood and cultured at 37°C for 36 to 48 hours for bacterial CFU enumeration. For colonization experiment, four to five mice per group were used. Muc5Ac^−/−^ mice were bred as a homozygous line as previously described (*51*). Muc5Ac^−/−^Muc5B^−/−^ double mutant mice were bred as a heterozygous line (due to the short life span of the homozygous mutant) and were genotyped as described (*52*).

For the transmission model, adult C3H/HeJ mice (6 to 8 weeks old, the Jackson Laboratory, female) in groups of five mice per cage were used. Two mice (donor) were inoculated with 5 μl of 100 CFU/μl bacteria, and the other three mice (recipient) were not inoculated. At 21 dpi, the nasal cavity of each mouse was excised, placed in 1 ml of cold PBS, homogenized, and plated as above to determine CFU.

In addition to the adult model, a neonatal transmission model (*42*) was further modified and developed. C57BL/6 mice (4 to 12 weeks, the Jackson Laboratory) were mated in pairs. The males were removed after pregnancy. The mothers normally give birth to 4 to 12 neonatal mice. Two to four neonatal mice at 5 days old (donor) were inoculated with 5 μl of 100 CFU/μl bacteria, and the remaining mice (recipient) were left uninoculated. Mice were euthanized at 21 dpi, and the nasal cavity was processed, and CFU was enumerated as above. For transmission experiment, three to five cages of mice (6 to 8 index mice, 8 to 15 naïve mice) were used.

### Isolation, growth, and differentiation of primary airway epithelia

Eight C57BL/6 mice (6 to 10 weeks, the Jackson Laboratory, male or female) were euthanized, and the exterior of the mice’s body was sanitized with 70% ethanol, during which precautions were taken to prevent ethanol from leaking into the nose. In a biosafety hood, the mice were dissected; the trachea, bronchi, and lungs were removed; and the trachea and bronchi were separated (*38*). Nasal septa tissues were dissected and separated following the procedure described previously (*40*). The nasal and tracheobronchial derived tissues (airway tissues) were placed in Falcon tubes containing cold collection media [Dulbecco’s modified Eagle’s medium (DMEM)–F12 supplemented with penicillin (100 IU/ml) and streptomycin (100 μg/ml), Pen/Strep] before proceeding to the next step. Mouse airway tissues were placed in 20 ml of cold dissociation medium [minimum essential medium supplemented with Pen/Strep, gentamycin (50 μg/ml), amphotericin B (50 ng/ml), and pronase (1.4 mg/ml)] for digestion. Tracheobronchial tissues were digested at 4°C 12 to 14 hours. The digestion of nasal septa tissues was monitored constantly by a phase contrast microscope, and often a digest at 37°C for 1.5 to 2 hours gave 90% release of nasal epithelia. Digestion was stopped by the addition of fetal bovine serum (FBS) to a final 10% concentration. The media containing released epithelia were set aside and kept at 4°C. The remaining tissues were then transferred to a petri dish containing 10 ml of culture medium (DMEM-F12 supplemented with Pen/Strep; gentamycin (50 μg/ml); amphotericin B (50 ng/ml); 5% FBS; and universal culture supplement (120 IU/ml), ITS] and gently washed (glass pipette) three times to release remanent epithelia. This washed media and the media from 4°C were combined (containing airway epithelia) and centrifuged (500*g*, room temperature, 5 min). The supernatant was discarded, and the cell pellet was gently resuspended with culture medium (prewarmed at 37°C) and evenly split between two petri dishes precoated with 500 μg of rat tail collagen per dish. The petri dishes were placed at 37°C (5% CO_2_) for 4 hours to selectively remove fibroblasts upon binding to collagen. Then, the petri dishes were gently swirled three times, and culture media containing the unbound cells were transferred to a Falcon tube. After centrifugation (500*g*, room temperature, 5 min), the supernatant was discarded, and the cell pellet was resuspended in mTEC+ medium [DMEM-F12 supplemented with Pen/Strep, amphotericin B (50 ng/ml), 5% heat-inactivated FBS, 200 mM GlutaMAX, insulin (10 μg/ml), transferrin (5 μg/ml), cholera toxin (100 ng/ml), epidermal growth factor (25 ng/ml), bovine pituitary extract (30 ng/ml), and 50 nM retinoic acid] and divided between two T75 cell culture flasks precoated with rat tail collagen (500 μg per flask). Following 3 days (37°C, in 5% CO_2_ chamber), fresh mTEC+ was changed every 2 days until mouse airway epithelia reached 90 to 100% confluency as determined by microscope.

At 100% confluency, epithelial cells were digested by trypsin-EDTA solution (0.5 mg/ml; Sigma-Aldrich) for 20 min to release epithelia. Following trypsin neutralization, wash, and centrifugation, cells were resuspended in mTEC+ medium and transferred to the apical chamber (insert) of a transwell (0.33 cm^2^; Corning) (30,000 cells per insert). mTEC+ medium was also added to the basolateral chambers. After 3 days postseeding, ~90% of cells were attached to the insert membranes. Both apical and basal media were removed, and fresh mTEC+ medium was added every 2 days. The TEER of mouse airway epithelia was monitored by an EVOM3 probe (World Precision Instruments) according to the instrument manual.

The TEER of each transwell was calculated using the equation below

$${\text{TEER}}_{\text{calculated}}=\left({\text{TEER}}_{\text{measured}}-{\text{TEER}}_{\text{blank}}\right)\times \text{surface area}$$

where ${\text{TEER}}_{\text{measured}}$ is the resistance ohms (Ω) measured by EVOM3 probe, ${\text{TEER}}_{\text{blank}}$ is the resistance ohms (Ω) of a blank well without epithelia, and the surface area is 0.33 cm^2^.

For mouse tracheal epithelia (MTE), cells were maintained with mTEC+ at both apical and basal chambers until TEER reached above 200 ohm∙cm^2^ (2 to 3 weeks). Then, the apical medium of MTE was removed and exposed to air (5% CO_2_ chamber), and the basal medium was switched to differentiation media (ALI media) [DMEM-F12 supplemented with Pen/Strep (100 μg/ml), amphotericin B (50 ng/ml), 2% NuSerum, and 50 nM retinoic acid]. Mature MTE (15 to 60 days post-ALI) were used for infection.

For mouse nasal epithelia (MNE), cells were maintained with mTEC+ at both apical and basal chambers for 12 days (TEER, 300 to 2000 ohm.cm^2^). Then, MNE cells were switched to ALI for 6 days (TEER drop by 90%). A second 12-day growth stage with mTEC+ at both apical and basal chambers was required to achieve 100% confluency post the first ALI. A second switch to ALI gave rise to fully differentiated MNE (TEER, 150 to 800 ohm.cm^2^). Mature MNE (15 to 60 days post the second ALI) were used for infection.

Primary NHBE cells were cultured to differentiate into a pseudostratified columnar epithelium containing ciliated and goblet cells as seen in vivo. The detailed methods were described in a prior study (*53*).

### Immunostaining and confocal microscopy

Mouse epithelial cells in trans-wells inserts were fixed in 4% paraformaldehyde, permeabilized in 0.5% Triton X-100 PBS, and washed three times with 0.1% PBST (PBS with 0.1% Tween 20). Inserts were blocked with 10% goat serum and incubated with primary antibodies for cilia [anti–beta tubulin antibody mouse monoclonal directly conjugated to CY3 (Sigma-Aldrich, C4585)] and mucin (Muc5Ac unconjugated rabbit polyclonal antibody (Bioss Inc., bs-7166R)]. Primary antibodies were incubated overnight in the dark at 4°C on a plate rocker. Inserts were then washed 3 times with 0.1% PBST and incubated with secondary antibody [Alexa Fluor 488 goat anti-rabbit (Thermo Fisher Scientific, A-11008)]. Inserts were washed three times with 0.1% PBST and incubated for F-actin [Alexa Fluor 647 Phalloidin (Thermo Fisher Scientific, A22287)]. Inserts were then washed three times with 0.1% PBST and wet-mounted on slides with SlowFade Gold Antifade Mountant with Dapi (Thermo Fisher Scientific, S36938). Images of cultures cells were acquired using a Nikon confocal microscope via cross-sectional *Z*-plane imaging with a 60× objective and 2× optical zoom. Files were saved in the Nikon Nd2 file format, and image analysis of inserts were conducted with the NIS-Elements viewer version 4.2. Images were then exported in JPEG format.

### Quantification of Muc5Ac by ELISA

Mouse nasal and tracheobronchial epithelial culture at day 15 post-ALI (for nasal epithelia, day 15 post the second round of ALI) were not washed until day 20. Then, 200 μl of HBSS was added to the apical surface of epithelial, and cells were incubated at 37°C for 15 min. Then, the HBSS was gently resuspended by a micropipette for three times, and all liquid (mucus in HBSS) on the apical surface was collected and frozen at −20°C until the analyses. The level of Muc5Ac was quantified by NOVUS mouse Muc5Ac ELISA kit (colorimetric) according to the instruction.

### Purification of PSM

PSM was further purified from the commercial porcine stomach mucus (Sigma-Aldrich). A 2 g of crude porcine stomach mucus was suspended in 40 ml of ultrapure water and dissolved by stirring at 4°C overnight. The suspension was centrifuged at 10,000*g*, 4°C, 1 hour, and the top 38 ml of supernatant was carefully transferred to a new centrifugation tube. The supernatant was recentrifuged (10,000*g*, 4°C, 1 hour), and the top 36 ml of supernatant was transferred to another tube and recentrifuged for the third time. The resulting top viscous, yellow-colored supernatant (35 ml) was frozen and lyophilized to dryness, and HBBS was used to dissolve the mucin powder at 100 mg/ml. Following sample aliquot, the mucus was stored at −20°C.

### Infection of mouse primary epithelial cultures

Mature mouse nasal epithelia or tracheobronchial epithelia were changed into antibiotic free ALI medium and left unwashed at the apical surface to accumulate mucus for 1 week. Then, around 500 CFU of *B. bronchiseptica* (*Bb*) wild type, Δ*b-Cool*, or complemented Δ*b-Cool* in 50 μl of PBS were inoculated to the apical surface of epithelia. Infected epithelia were incubated at 37°C (5% CO_2_ chamber) for 2 hours, and then 200 μl of HBSS was added to the apical surface of infected cells or control uninfected cells, and 200 μl of apical wash solution was removed and plated for CFU. The infected epithelia were immediately put back to 37°C. Then, the apical wash was performed again at 6, 10, 24, 48, 72, and/or 96 hours postinoculation. At and beyond 24 hours postinoculation, TEER was measured after adding the 200 μl of HBSS and before withdrawing the 200 μl of apical wash. For infection experiments, four to five wells per group were used.

To enumerate epithelia-associated CFU, mouse nasal epithelia after apical wash were washed by 300 μl of HBSS for another twice, then 100 μl of HBSS was added to the apical surface, and cells were cooled on ice. A 100 μl of 0.2% Triton X-100/PBS was added to the apical surface, and cells were disassociated from the membrane by pipetting. The cell resuspensions were incubated for 5 min at room temperature until serial dilution and CFU enumeration.

For binding experiments with the addition of NHBE mucus, 25 μl of NHBE mucus was added to each well of epithelial cells before inoculation, and additional NHBE mucus was added after each wash (4 μl of NHBE mucus was added post–2-hour wash, and 8 μl was added post–6- and 10-hour wash).

The percent CFU bound to epithelia was calculated as

CFU associated with epithelia/(CFU in the apical wash + CFU associated with epithelia) × 100%.

Similarly, for MTE infection experiments with addition of external mucus, 25 μl of mucus (MNM or PSM) was added before inoculation, 4 μl was added post–2-hour wash, and 8 μl was added post–6- and 10-hour wash. MNM was directedly collected from the apical surface of mouse nasal epithelia. The concentration of PSM was adjusted to be equivalent to MNM based on total carbohydrate content quantified by a phenol-sulfuric acid assay (*54*).

### Mucus interaction assay

A 150 μl of ovalbumin (1 mg/ml in PBS), MNM, or PSM (equivalent to MNM in total carbohydrate content) was added to a 96-well plate and dried at 37°C for 48 hours. The plate was washed with PBS six times before inoculation, with ~500 CFUs of *Bb* wild type or Δ*b-Cool* in 100 μl of PBS. The plate was incubated at 4° or 37°C for 1 hour and then immediately placed on ice. Another 100 μl of PBS was added to each well and gently resuspended three times, and then 100 μl of liquid was withdrawn and plated for CFUs.

### Statistical analyses

Statistical analyses of wild type and mutant CFUs were performed by an unpaired Student’s two-tailed *t* test. *P* value above 0.05 was considered not statistically significant and was either left unlabeled or indicated as “ns.” *P* value between 0.05 and 0.1 was occasionally displayed to indicate trends but was not considered statistically significant. *P* value between 0.01 and 0.05 was represented by *. *P* value between 0.001 and 0.01 was represented by **. *P* value between 0.0001 and 0.001 was shown as ***. *P* value below 0.0001 was shown as ****. All bars in the figures represent mean value, and all error bars represent SEM.

## Supplementary Materials

### This PDF file includes:

Figs. S1 to S14 Tables S1 to S3 References
