# Supplementary material for: Bordetellae colonization oligosaccharide (b-Cool), a glycan crucial for nasal colonization
Source: Sci Adv. 2025 Sep 3;11(36):eadw7764. doi: 10.1126/sciadv.adw7764 (PMC12407052; doi:10.1126/sciadv.adw7764)
Supplement: Supplementary file 1 — Figs. S1 to S14 Tables S1 to S3 References [file sciadv.adw7764_sm.pdf]

Science Advances
AAAS

Supplementary Materials for

Bordetellae colonization oligosaccharide (b-Cool), a glycan crucial for nasal colonization

Yang Su *et al.*

Corresponding author: Maor Bar-Peled, peled@ccrc.uga.edu; Eric T. Harvill, harvill@uga.edu

Sci. Adv. **11**, eadw7764 (2025)
DOI: 10.1126/sciadv.adw7764

This PDF file includes:

Figs. S1 to S14
Tables S1 to S3
References

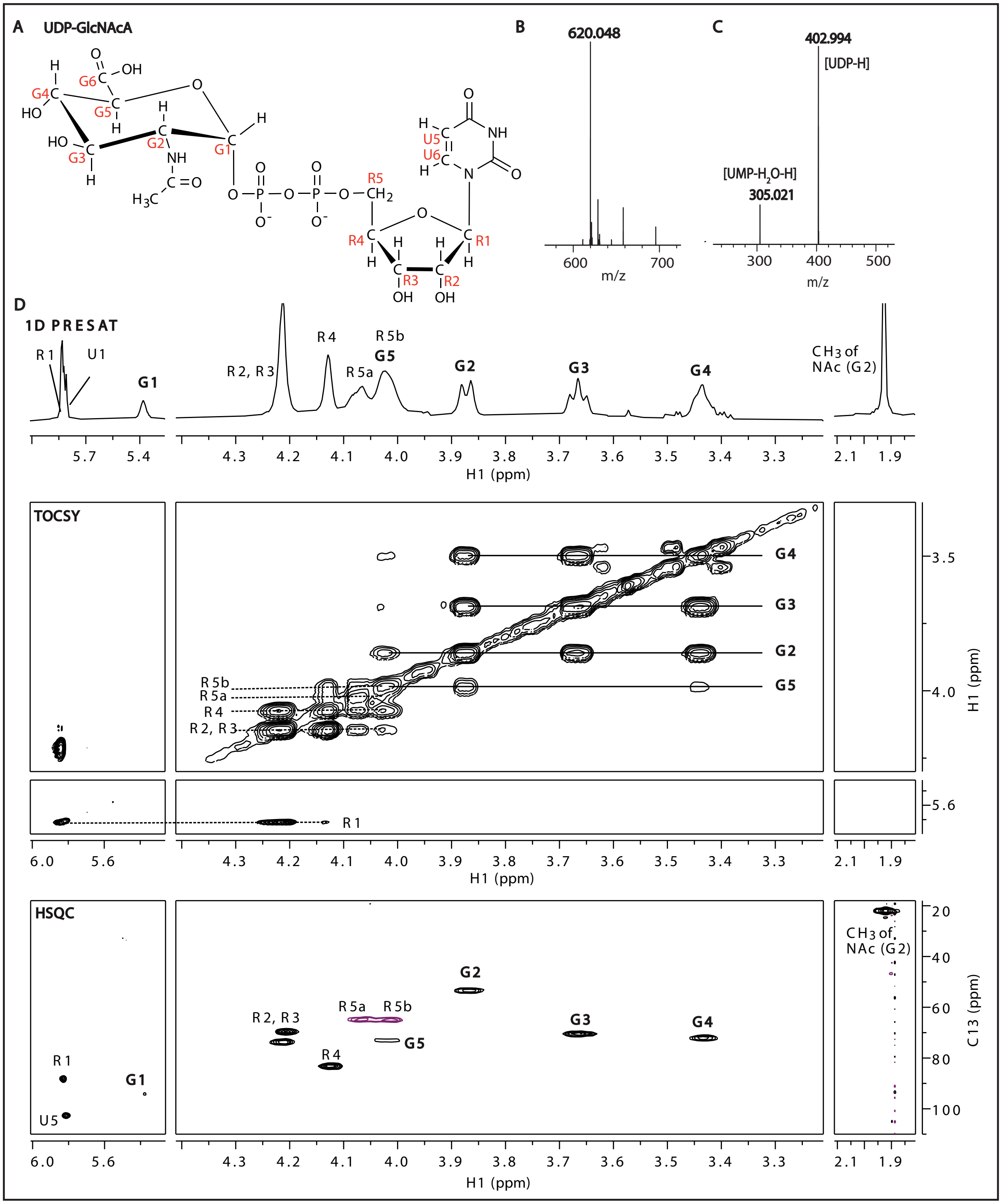

Fig. S1. NMR analyses identified the enzymatic product of BB2925 as UDP-GlcNAcA. The enzymatic product of BB2925 eluting at 15 minutes from the Hilic column had an m/z of 620.05 (**B**), with MS/MS fragmentation of 402.9 and 305.0 (**C**). The NDP-sugar was purified by a Q15 anionic exchange column, and its chemical structure was determined by 1D PRESAT NMR and 2D TOCSY, HSQC NMR experiments (600 MHz, 25°C) (**D**). These data provide evidence that the enzyme product is UDP-GlcNAcA (**A**), similar to the UDP-GlcNAcA isolated from *Bacillus cereus* (*55*).

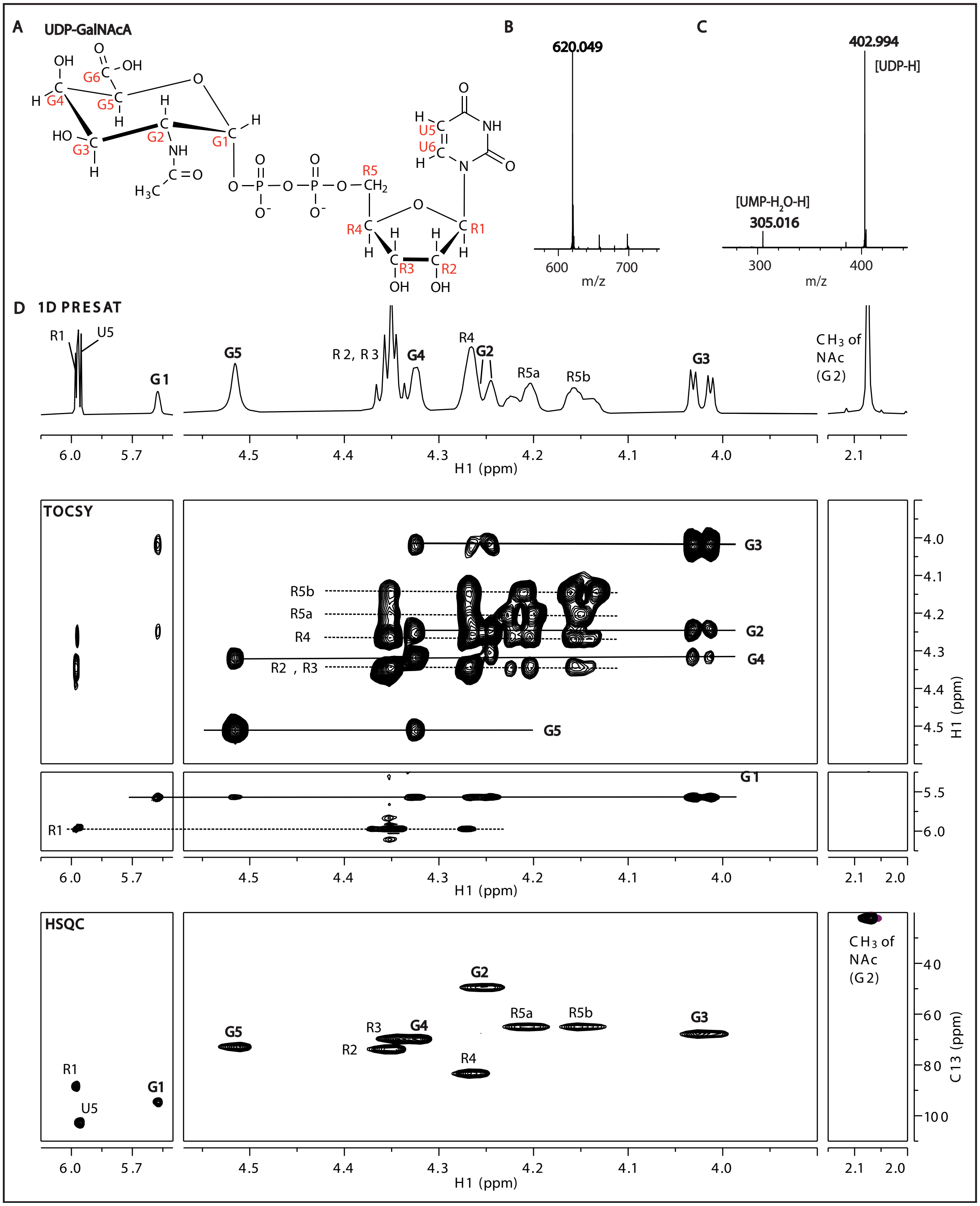

Fig. S2. NMR analyses identified the enzymatic product of BB2924 as UDP-GalNAcA. The enzymatic product of BB2924 eluting at 17 minutes from the Hilic column had an m/z of 620.05 (**B**), with MS/MS fragmentation of 402.9 and 305.0 (**C**). The NDP-sugar was purified by a Q15 anionic exchange column, and its chemical structure was determined by 1D PRESAT NMR and 2D TOCSY, HSQC NMR experiments (600 MHz, 25°C) (**D**), indicating that the enzyme product is UDP-GalNAcA (**A**).

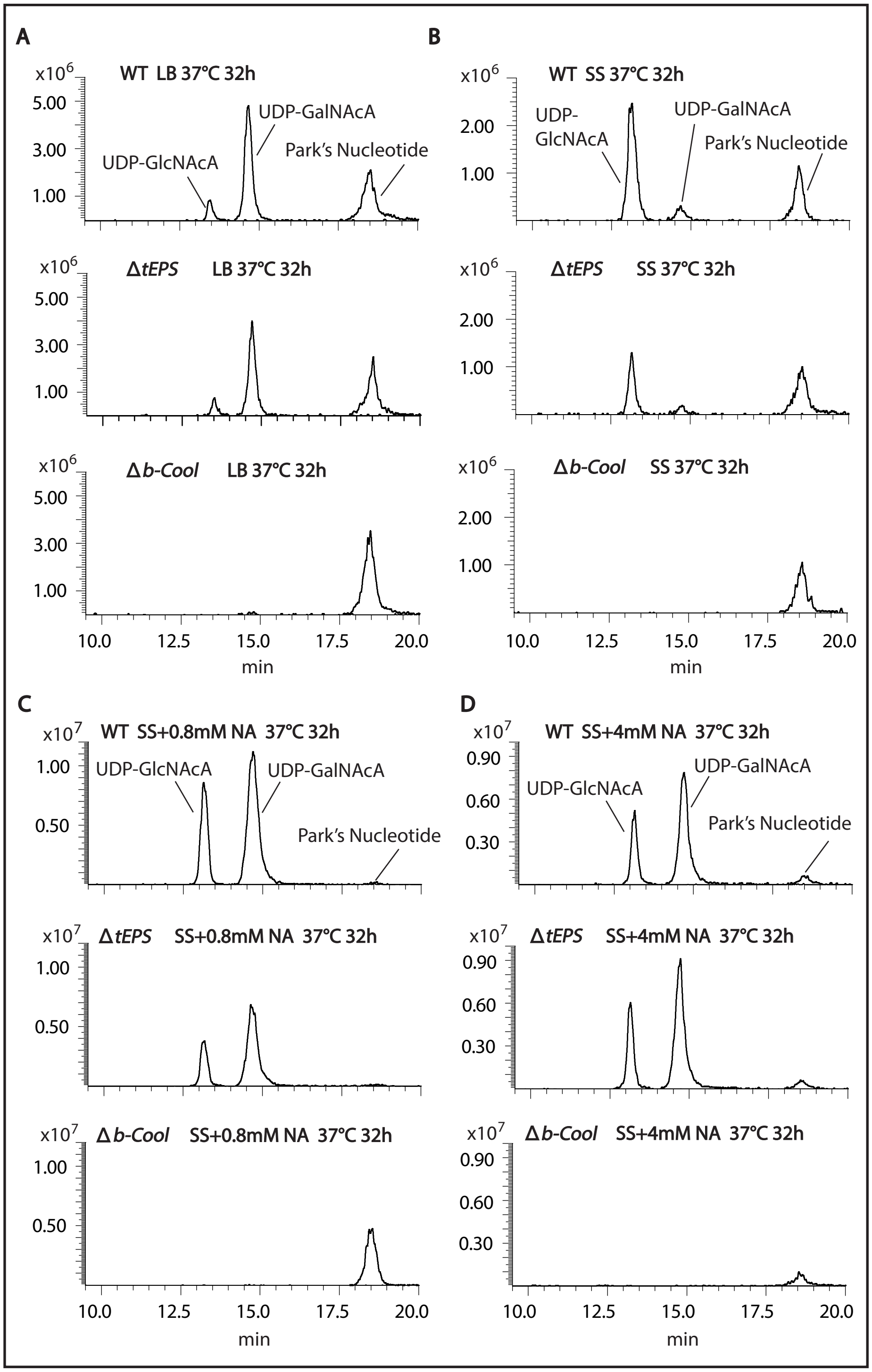

Fig.S3. LC-MS UDP-sugar profiles of WT, Δ*tEPS*, and Δ*b-Cool* in various growth media. Slightly reduced amounts of UDP-GlcNAcA and UDP-GalNAcA were observed in Δ*tEPS* when compared to WT in LB (A), SS (B), or SS supplemented with 0.8 mM nicotinic acid (NA) (C). Δ*tEPS* had similar UDP-sugar profiles as the WT when grown in SS supplemented with 4mM NA (D). In all growth media, no detectable amount of UDP-GlcNAcA and UDP-GalNAcA was observed in the Δ*b-Cool*. Note: When grown in SS media, different amounts of nicotinic acid were supplemented to stimulate different virulence states. Bacteria grew in SS media was in bvg plus

state (bvg^+). 0.8 mM NA modulated the bacteria in the bvg intermediate state (bvg^i), and 4 mM NA modulated the bacteria in the bvg minus state (bvg^-) (*56*).

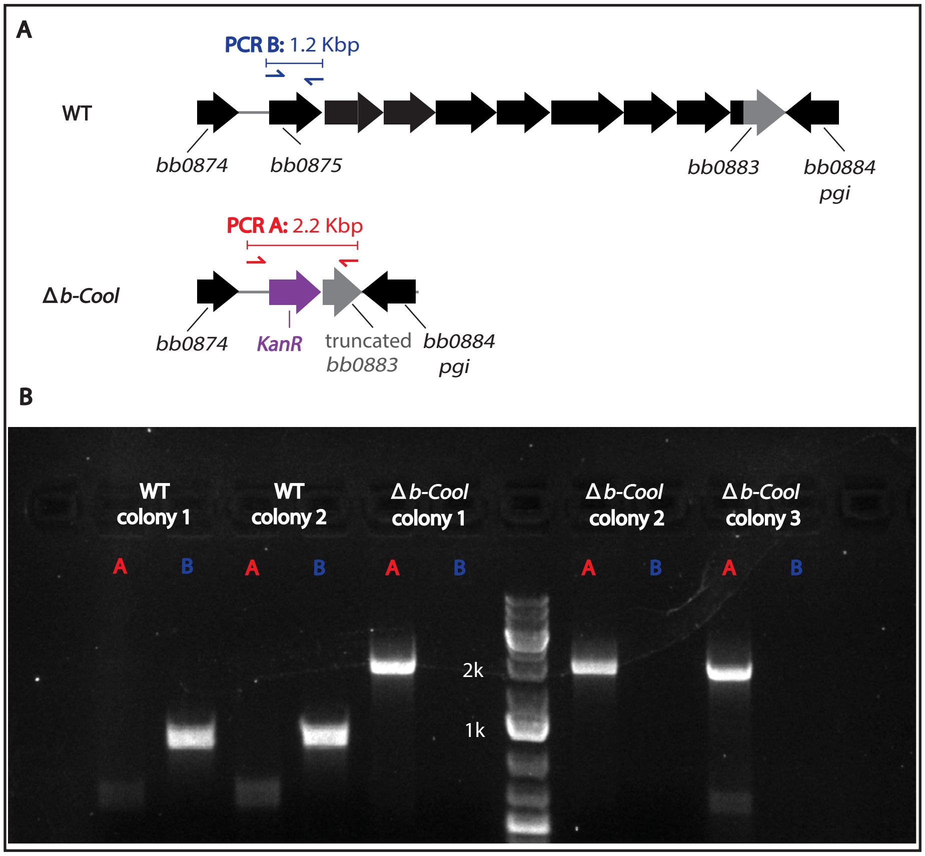

Fig. S4. Deletion of the 9-gene b-Cool locus by allele exchange. The 9-gene b-Cool locus region, starting from the beginning of *bb0875* till the middle of *bb0883*, was replaced by a kanamycin-resistant gene (*kanR*) by allele exchange (Panel **A**). The *bb0883* was truncated but not completely deleted to avoid interrupting its downstream gene *bb0884* (*pgi*), predicted to encode for the glucose-6-phosphate isomerase. Two pairs of primers were designed to verify the deletion of the b-Cool locus (Panel **B**), including one pair flanking the *kanR* and truncated *bb0883* region (PCR A) and another pair flanking the *bb0875* (PCR B). The Δ*b-Cool* is expected to be positive for PCR A but negative for PCR B, and vice versa for the wild type.

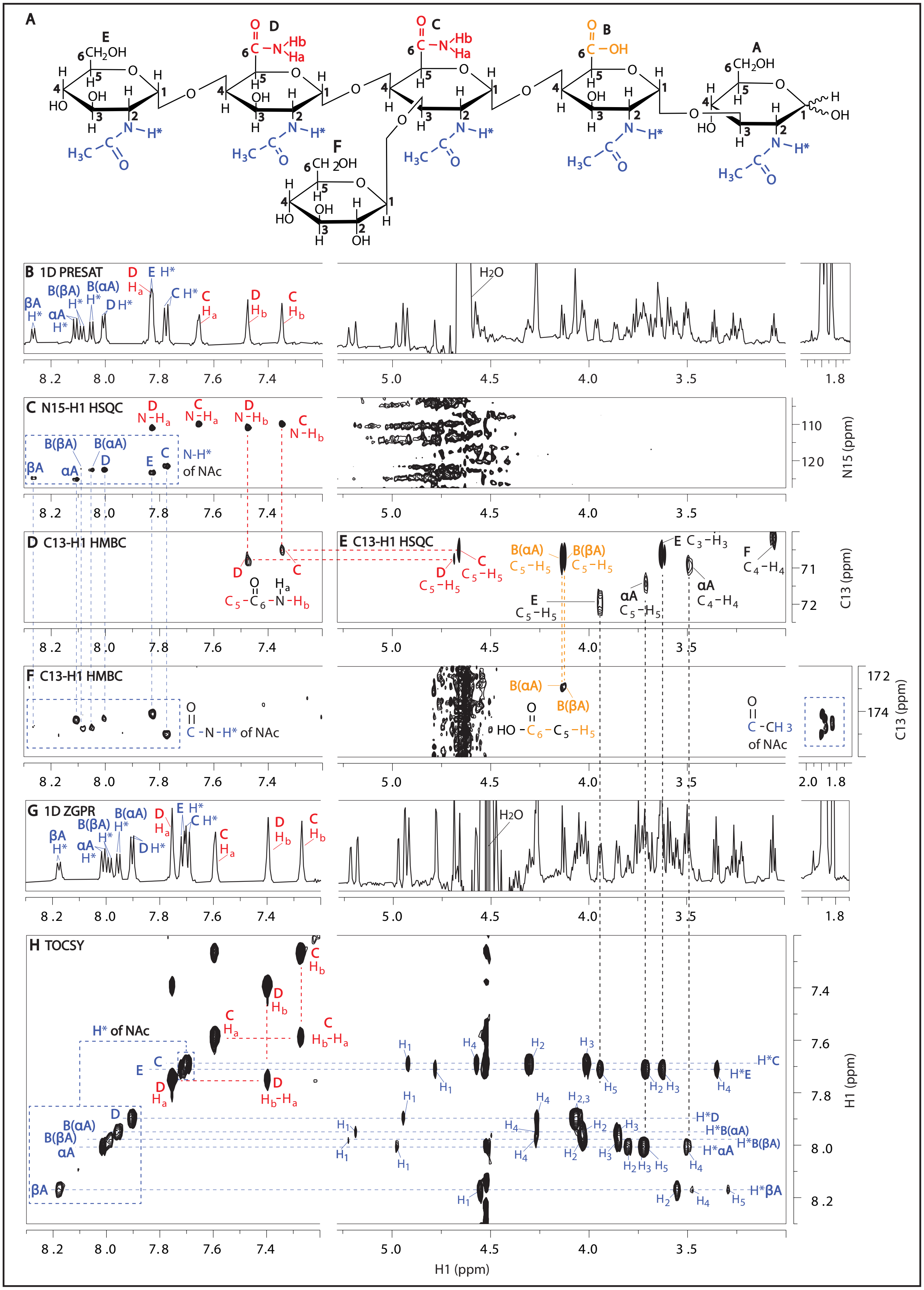
A
B 1D PRESAT
C N15-H1 HSQC
D C13-H1 HMBC
E C13-H1 HSQC
F C13-H1 HMBC
G 1D ZGPR
H TOCSY
N-H* of NAc
C-N-H* of NAc
HO-C6-C5-H5
C-CH3 of NAc
H* of NAc
N15 (ppm)
C13 (ppm)
H1 (ppm)

Fig. S5. NMR Characterization of amide protons of b-Cool distinguishing the uronate-amide (CO-NH2) group from the carboxylate (COOH) group. 800 MHz data of b-Cool in 85.5%H20/9.5%D2O/5% deuterated acetic acid at 25°C (panels **B-F**) and 35°C (panels **G-H**). Panel **A**) The chemical structure of m/z 1234.4 b-Cool glycan. Panel **B**) 1D proton showing amide proton signals from the two GalNAcAN -NH2 groups (C, D-NHa,b) and the five 2-NHAc groups (A-E). Panel **C**) ^{15}N-^{1}H HSQC spectrum showing N-H correlations for 6-NH2 groups of residues C and D, as well as 2-NHAc for residues A-E. The chemical shifts of ~110 ppm are consistent with -NH2 nitrogens, whereas the shifts of ~125 ppm are consistent with -NHAc nitrogens. Panel **D**) ^{13}C-^{1}H HMBC spectrum showing C-H correlation between C6-NH2 protons and C5 of the GalNAcAN residues C and D, consistent with C6 uronate-amide groups. Panel **E**) ^{13}C-^{1}H HSQC spectrum showing C-H correlations between H5 and C5 of the GalNAcAN residues C and D, confirming the carbon signals for C5. The horizontal red lines connect to the same C5 carbons in panel D. Panel **F**) ^{13}C-^{1}H HMBC spectrum showing C-H correlations between 2-NAc amide protons and their carbonyl carbons. They correspond to other correlations between the methyl protons (1.8-1.95 ppm) of the 2-NAc groups and the carbonyls. In addition, the C6 carboxylic carbon for residue B at ~173 ppm correlates with the H5 proton of B, identified in the HSQC of panel E. Correlations between the -NH2 protons and their respective carbonyls were not observed, probably due to small 2-bond coupling values. Panel **G**) 1D proton, as in panel B, except at 35°C. Panel **H**) 2D TOCSY showing the H-H connection between the geminal -NH2 protons of residues C and D. The connections (horizontal blue lines) to ring protons from the respective 2-NAc amide protons can be correlated with the assigned ring protons shown in panel E (vertical black lines).

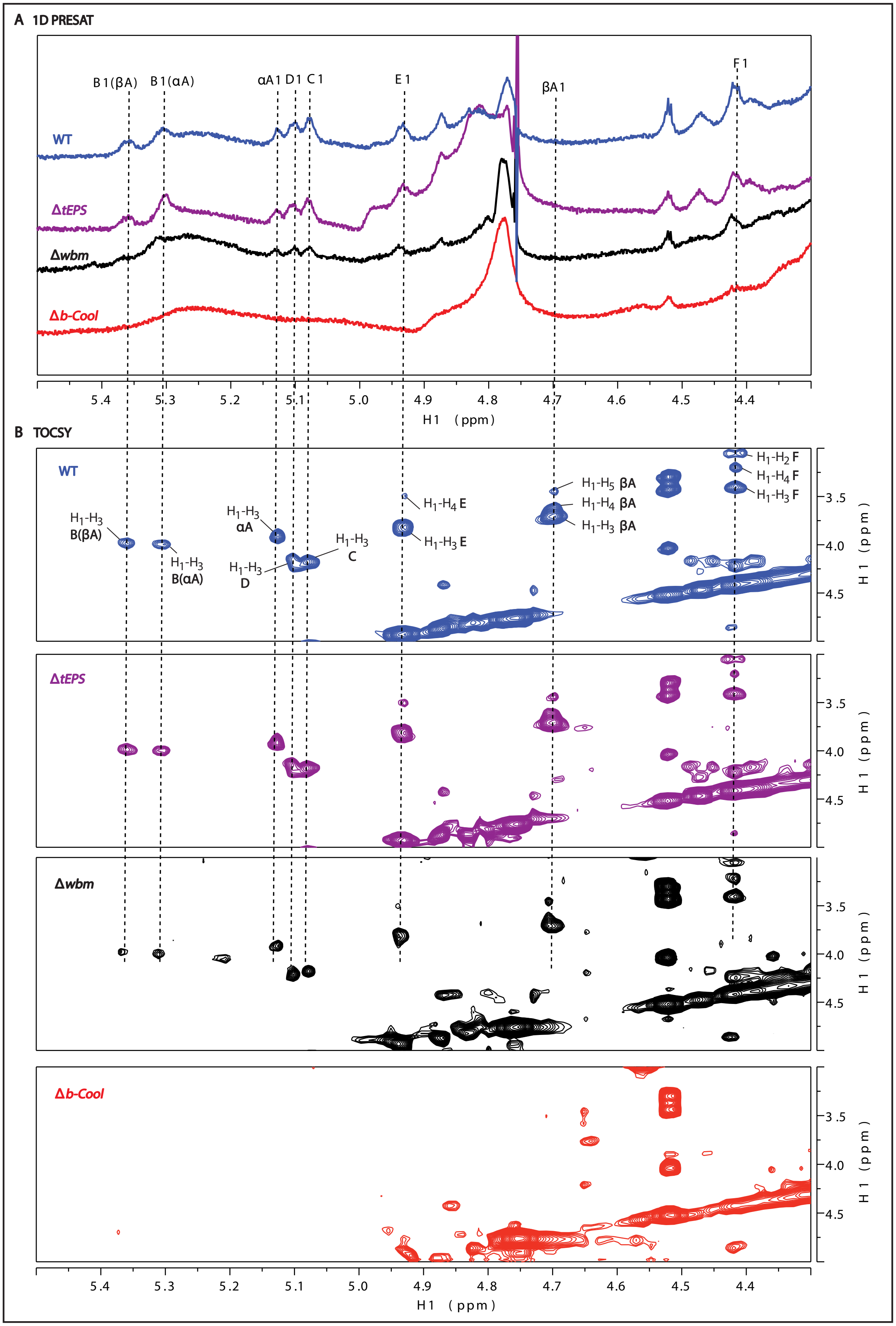

A 1D PRESAT
B1(βA)
B1(αA)
αA1
D1
C1
E1
βA1
F1
WT
ΔtEPS
Δwbm
Δb-Cool
5.4
5.3
5.2
5.1
5.0
4.9
4.8
4.7
4.6
4.5
4.4
H1 (ppm)
B TOCSY
WT
H1-H3 B(βA)
H1-H3 B(αA)
H1-H3 αA
H1-H3 D
H1-H3 C
H1-H4 E
H1-H3 E
H1-H5 βA
H1-H4 βA
H1-H3 βA
H1-H2 F
H1-H4 F
H1-H3 F
ΔtEPS
Δwbm
Δb-Cool
3.5
4.0
4.5
H1 (ppm)

Fig. S6. Magic Spin NMR Analyses show b-cool structure in intact cells. *B. bronchiseptica* wild type, Δ*tEPS*, Δ*wbm*, and Δ*b-Cool* were cultured, washed, and resuspended in PBS/D_2O and analyzed by HR-MAS 1D PRESAT (**A**) and 2D TOCSY (**B**) NMR (600 MHz, 25°C). The peaks and cross-peaks (represented by vertical dash lines) of b-Cool glycan were consistent with the purified structure described in table S1.

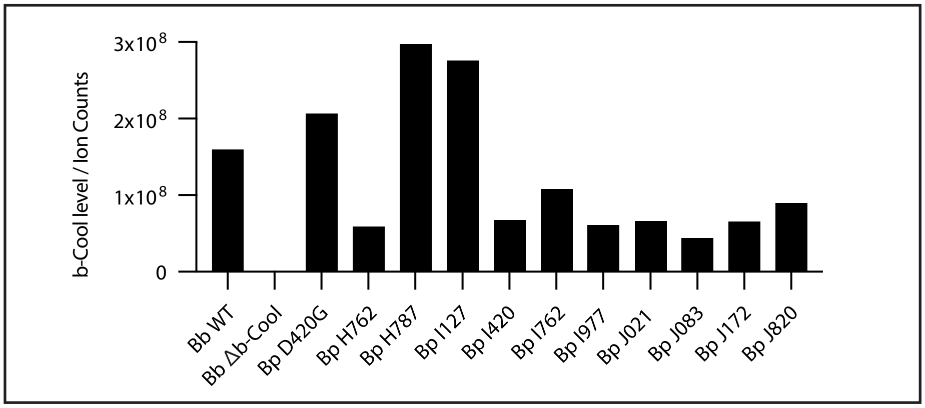

Fig. S7. Detection of b-Cool in 11 recent *B. pertussis* clinical isolates. *B. pertussis* clinical isolates were cultured for 2 days in SS medium at 37°C, and metabolites were extracted and then analyzed by LC-MS/MS. The b-Cool (m/z 1234.4) glycan was detected in all 11 *B. pertussis* clinical isolates.

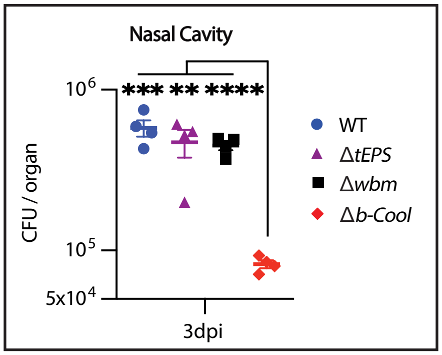

Fig. S8. The nasal colonization defect was unique to Δ*b-Cool*.
Around 500 CFU of *B. bronchiseptica* wild type, Δ*tEPS*, Δ*wbm*, or Δ*b-Cool* were delivered intranasally to C57BL/6 mice. Mice were sacrificed at 3 dpi, and the nasal cavity CFU level was examined. Δ*tEPS* and Δ*wbm* showed similar CFU as the wild type. Δ*b-Cool* showed ~90% fewer CFU compared to the wild type, Δ*tEPS*, or Δ*wbm*.

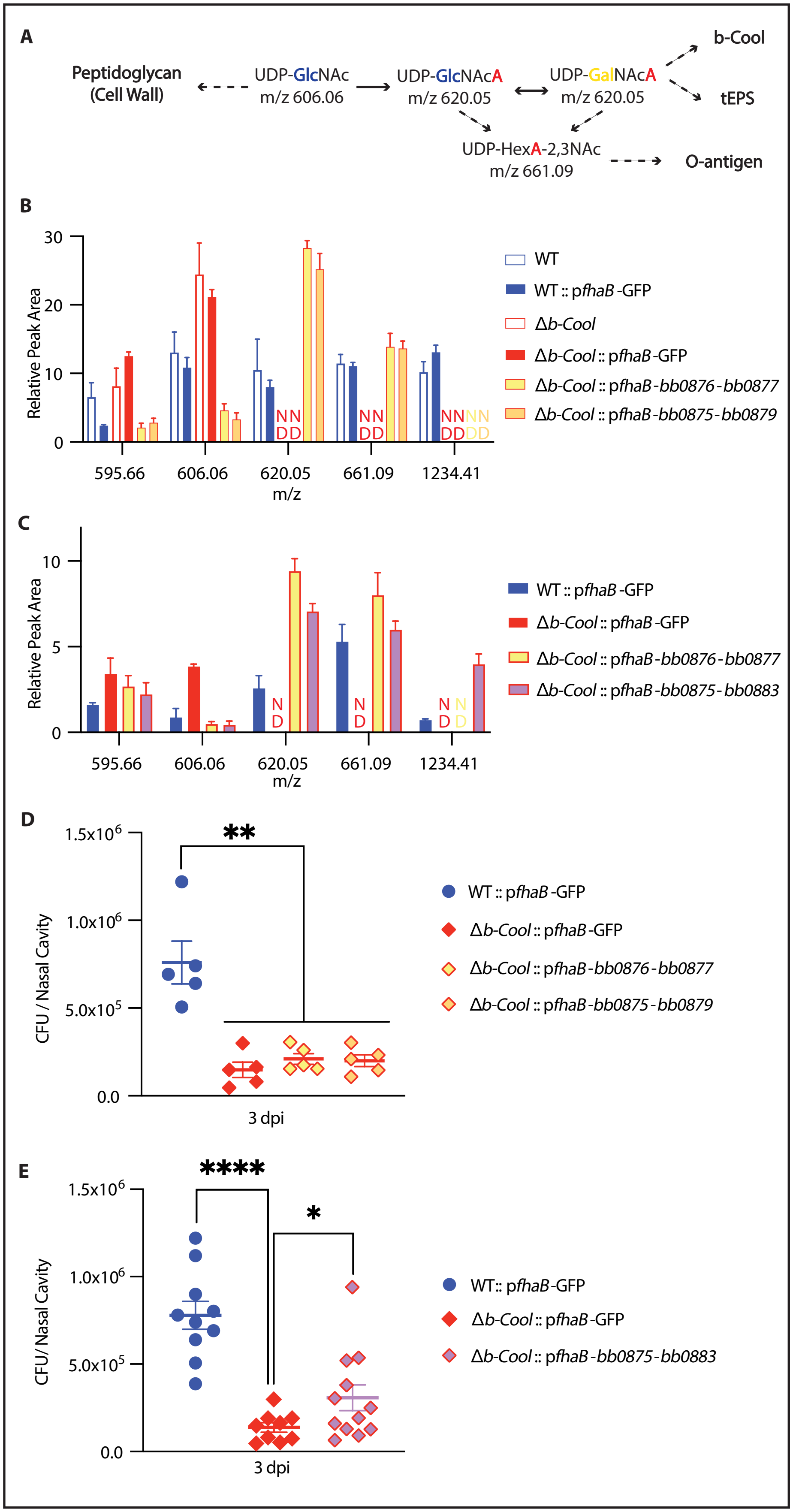

A
Peptidoglycan
(Cell Wall)
UDP-GlcNAc
m/z 606.06
UDP-GlcNAcA
m/z 620.05
UDP-GalNAcA
m/z 620.05
b-Cool
tEPS
UDP-HexA-2,3NAc
m/z 661.09
O-antigen
B
Relative Peak Area
30
20
10
0
595.66
606.06
620.05
661.09
1234.41
m/z
ND
WT
WT :: pfhaB-GFP
Δb-Cool
Δb-Cool :: pfhaB-GFP
Δb-Cool :: pfhaB-bb0876-bb0877
Δb-Cool :: pfhaB-bb0875-bb0879
C
Relative Peak Area
10
5
0
595.66
606.06
620.05
661.09
1234.41
m/z
ND
WT :: pfhaB-GFP
Δb-Cool :: pfhaB-GFP
Δb-Cool :: pfhaB-bb0876-bb0877
Δb-Cool :: pfhaB-bb0875-bb0883
D
CFU / Nasal Cavity
1.5x10^6
1.0x10^6
5.0x10^5
0.0
**
3 dpi
WT :: pfhaB-GFP
Δb-Cool :: pfhaB-GFP
Δb-Cool :: pfhaB-bb0876-bb0877
Δb-Cool :: pfhaB-bb0875-bb0879
E
CFU/ Nasal Cavity
1.5x10^6
1.0x10^6
5.0x10^5
0.0
****
*
3 dpi
WT:: pfhaB-GFP
Δb-Cool :: pfhaB-GFP
Δb-Cool :: pfhaB-bb0875-bb0883

Fig. S9. The nasal colonization defect of Δ*b-Cool* was not due to disruption of NDP-sugar flux. The biosynthesis pathways of multiple complex glycans (peptidoglycan, lipopolysaccharide LPS, tEPS, b-Cool) are interconnected (**A**). NDP-sugar flux in Δ*b-Cool* was restored to WT level by both two-gene and 5-gene complementations (pBBR-*bb0876*-*bb0877*, pBBR-*bb0875*-*bb0879*) (**B**). ND: Not detected. The 9-gene complementation (pBBR-*bb0875*-*bb0883*) restored the production of b-Cool (m/z 1234.4) (**C**). The nasal colonization of WT, Δ*b-Cool* as well as complemented Δ*b-Cool* (pBBR-*bb0876*-*bb0877*, pBBR-*bb0875*-*bb0879*, pBBR-*bb0875*-*bb0883*) was determined at 3dpi (**D, E**).

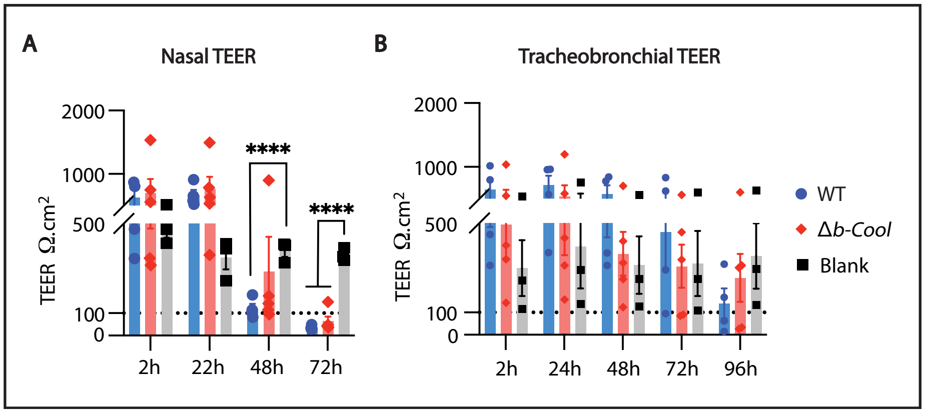

Fig. S10. Trans-epithelial electrical resistance (TEER) during mouse primary nasal epithelia infection (A) and mouse primary tracheobronchial epithelial infection (B). The electrical resistance of epithelial cells was deducted from the resistance of a blank trans-well membrane. Epithelial cells with full integrity had a resistance of above or around 100 $\Omega.cm^2$. A drastic drop in TEER to below 100 $\Omega.cm^2$ was observed 48h post-inoculation of nasal epithelia and 96h post-inoculation of tracheobronchial epithelia.

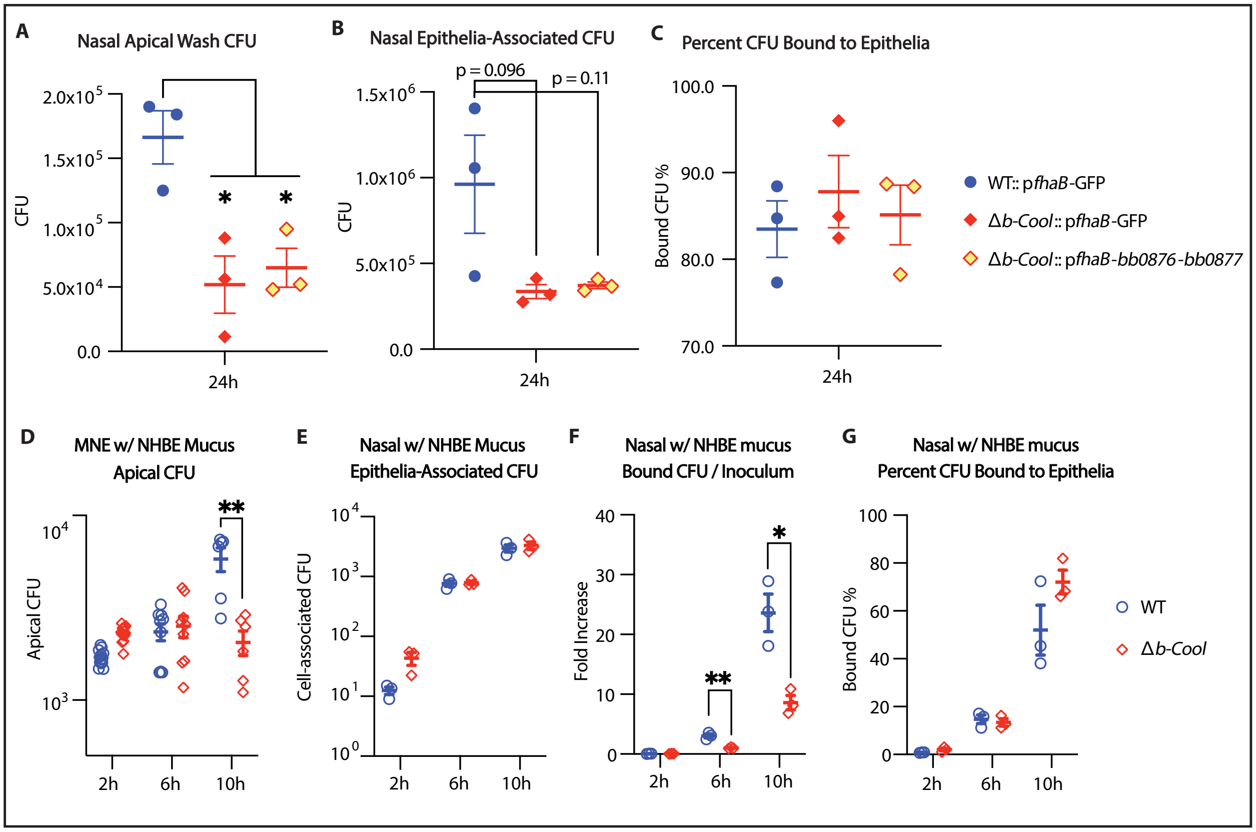

Fig. S11. Δ*b-Cool* showed no defect in binding to mouse nasal epithelial cells.
Two individual experiments were conducted to compare the binding of WT and Δ*b-Cool* to mouse nasal epithelia. In the first experiment (A-C), similar amount of *Bb* wild type, Δ*b-Cool*, or the 2-gene complemented Δ*b-Cool* were inoculated to primary mouse nasal epithelial cultured in an air-liquid interface similarly as Fig.4. At 24h post inoculation, bacteria CFU from apical wash and associated with nasal epithelia were enumerated. Δ*b-Cool* and the 2-gene complemented Δ*b-Cool* had less CFU than the wild type in both apical wash and epithelia-associated populations (**A, B**). The wild type, Δ*b-Cool*, and 2-gene complemented Δ*b-Cool* had similar ability to bind to mouse nasal epithelial cells (**C**). To reduce the variation of experiment likely due to varied level of mucus secretion, a second experiment was conducted (D-G), where primary mouse nasal epithelial cells were supplemented with additional mucus from normal human bronchial epithelial cells (NHBE) before inoculation and after each apical wash. Despite 3 times more CFUs of Δ*b-Cool* than the wild type was inoculated to the apical of nasal epithelia, at 10h post inoculation, Δ*b-Cool* was found to have significantly less CFU than the wild type in the apical wash (D). In addition, after adjusting the inoculum, there is also less Δ*b-Cool* than wild type in the epithelia-associated population at 6h and 10h post inoculation (F), while the relative percent of bacteria bound to epithelia was similar for wild type and Δ*b-Cool* (G).

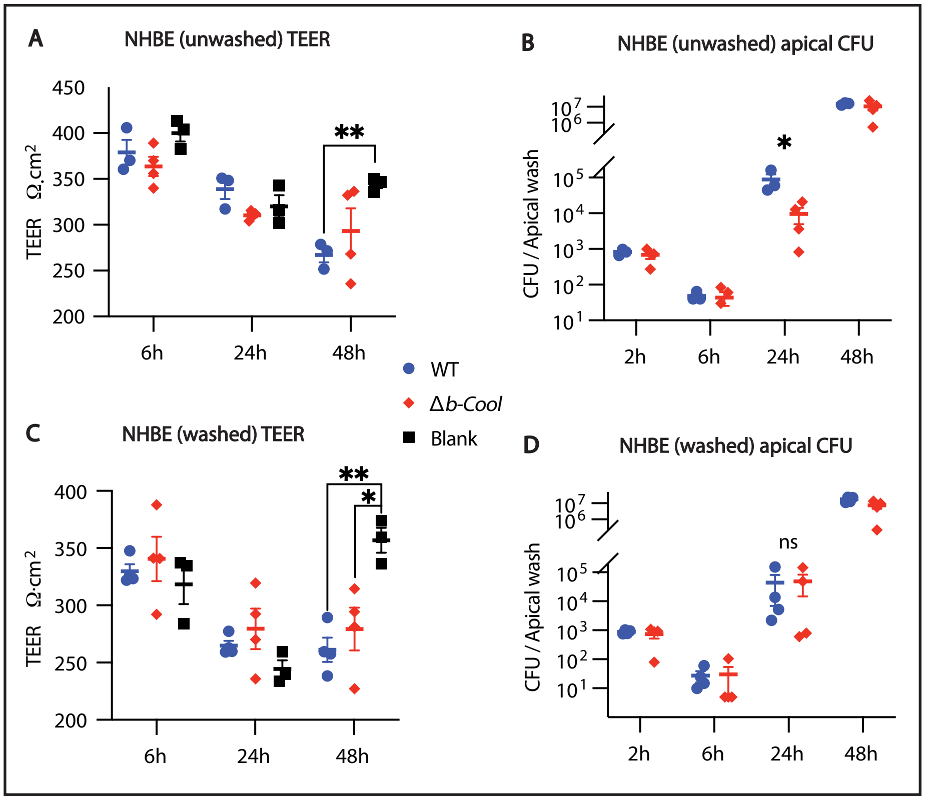

Fig. S12. Δ*b-Cool* showed colonization defect in normal human bronchial epithelia (NHBE) ALI in presence of mucus. Normal human bronchial epithelia were harvested from human donors and cultured at the ALI. A thick mucus layer was observed on the apical surface of the healthy NHBE culture. The apical surface of NHBE was either unwashed or washed with HBSS before inoculation with WT or Δ*b-Cool*. In both experiments, the TEER dropped 48h post-infection compared to the uninfected control (A, C). A drastic colonization defect of Δ*b-Cool* was observed in unwashed NHBE with accumulation of mucus before inoculation, while Δ*b-Cool* showed no statistical difference with WT in colonization in washed NHBE without accumulation of mucus layer (B, D).

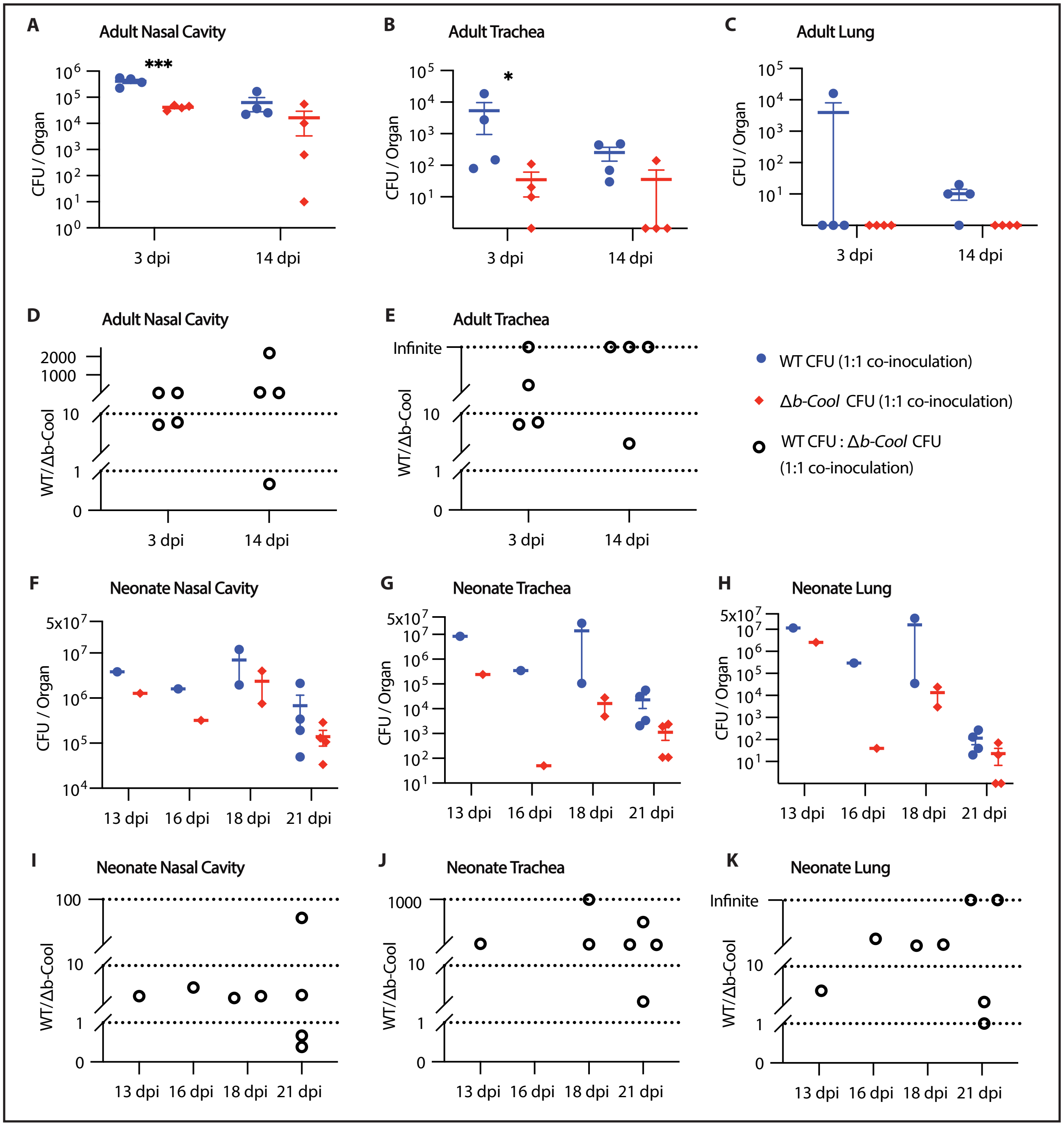

Fig. S13. Δ*b-Cool* had persistent defects across respiratory organs when co-inoculated with the wild type at a 1:1 ratio. Around 500 CFU of wild type and Δ*b-Cool* mixed at a 1:1 ratio was delivered intranasally to adult C57BL/6 mice of 6-8 weeks old. Mice were sacrificed periodically at 3 and 14 dpi, and the respiratory organs were excised. The CFU level of each strain was examined by plating on BG plates with different antibiotic selections, as shown in A (nasal cavity), B (trachea), and C (lung). The ratio of wild type to Δ*b-Cool* in each mouse was shown in D (nasal cavity) and E (trachea). The infinite ratio was due to no detection of Δ*b-Cool* CFU. In the neonatal competition transmission experiment with similar co-inoculation of wild type and Δ*b-Cool* at a 1:1 ratio, the CFU of wild type and Δ*b-Cool* in the respiratory organs of donor mice was examined at the end of the transmission experiment (21dpi) or when the donor mice were found moribund in the middle of experiments (13, 16, 18 dpi). The CFU of the wild type and Δ*b-Cool* were shown

in F (nasal cavity), G (trachea), and H (lung). The ratio of wild type to Δ*b-Cool* in each neonatal mouse was shown in I (nasal cavity), J (trachea), and K (lung).

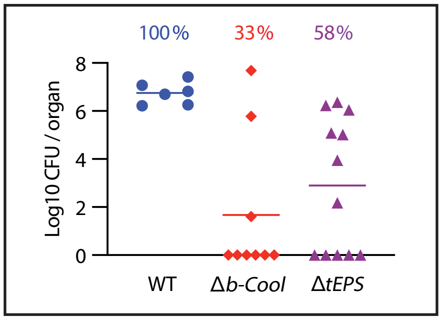

Fig. S14. Δ*b-Cool* show more severe transmission defect compared to Δ*tEPS*. An adult transmission model was used to compare the relative ability of WT, Δ*b-Cool*, and Δ*tEPS* to transmit amongst mice. Per cage of C3H/HeJ mice, two donor mice were inoculated with 500 CFU of WT, Δ*b-Cool*, or Δ*tEPS* and then co-housed with three recipient mice. Nasal cavity CFUs of recipient mice at 21 dpi were compared. WT showed transmission to 100% of the recipient mice. Δ*b-Cool* showed transmission to 33% (3 out of 9) of the recipient mice, similar to what we observed from the neonatal transmission model (Fig. 5). Δ*tEPS* exhibited 58% transmission rate, which is almost twice of Δ*b-Cool*.

Table S1. ^{1}H and ^{13}C NMR data of m/z 1234.4 glycan.
The m/z 1234.4 glycan was purified by Seppak C18 (eluted with water) and gel chromatography on Sephadex G-15 or Q15 anionic exchange, followed by gel filtration on BioGel P2. The chemical structure of m/z 1234.4 glycan was solved by 1D and 2D NMR (500 MHz 40° C). The H1 and C13 chemical shift of each residue are shown below.

**Unit, Compound**	Atom	1	2	3	4	5	6
α-A, GlcNAc	^{1}H	5.14	3.96	3.91	3.65	3.87	3.82; 3.87
	^{13}C	92.2	53.8	79.4	72.2	72.8	61.5
β-A, GlcNAc	^{1}H	4.72	3.73	3.73	3.64	3.46	3.74; 3.91
	^{13}C		56.5	81.0	72.2	77.0	61.7
B->α-A, α-GalNAcA	^{1}H	5.34	4.20	4.00	4.41	4.20	
	^{13}C	99.3	50.6	67.8	80.0	72.4	
B->β-A, α-GalNAcA	^{1}H	5.38	4.20	4.00	4.41	4.18	
	^{13}C	99.4	50.6	67.8	80.0	72.4	
C, α-GalNAcAN	^{1}H	5.10	4.48	4.17	4.74	4.83	
	^{13}C	100.3	49.7	76.9	75.0	71.8	
D, α-GalNAcAN	^{1}H	5.11	4.24	4.21	4.44	4.87	
	^{13}C	97.5	50.5	67.5	77.0	72.0	
E, α-GlcNAc	^{1}H	4.95	3.88	3.79	3.51	4.11	3.76; 3.78
	^{13}C	98.8	54.9	71.8	70.9	73.2	61.4
F, β-Glc	^{1}H	4.44	3.07	3.43	3.23	3.40	3.66; 3.91
	^{13}C		74.1	76.8	71.3	76.9	62.5

Table S2. Plasmid constructs generated in this study.

Plasmid Construct Name	Antibiotic Selection	Purpose of construct
pET28b-p*T7-bb2925*	Kanamycin	Expression of BB2925 in *E. coli*. Induced by 0.5 mM IPTG.
pCDF-p*araB-bb2924*	Spectinomycin	Expression of BB2924 in *E. coli*. Induced by 6.6 mM L-arabinose.
pCDF-p*araB-bb2925-bb2924*	Spectinomycin	Co-expression of BB2925 and BB2924 in *E. coli*. Induced by 6.6 mM L-arabinose.
pCDF-p*araB-bb0876-bb0877*	Spectinomycin	Co-expression of BB0876 and BB0877 in *E. coli*. Induced by 6.6 mM L-arabinose.
pEXΔ*b-Cool*	Kanamycin	Allele exchange deletion of 9-gene b-Cool locus. Counter selection induced by 15% sucrose.
pBBR-p*fhaB*-GFP	Gentamycin	Δ*b-Cool* complementation plasmid blank control.
pBBR-p*fhaB-bb0876-bb0877*	Gentamycin	Δ*b-Cool* complementation plasmids expressing two NDP-sugar synthases driven by a *fhaB* promoter.
pBBR-p*fhaB-bb0875-bb0879*	Gentamycin	Δ*b-Cool* complementation plasmids expressing five genes of b-Cool locus driven by a *fhaB* promoter.
pBBR-p*fhaB-bb0875-bb0883*	Gentamycin	Δ*b-Cool* complementation plasmids expressing the entire 9-gene b-Cool locus driven by a *fhaB* promoter.

Table S3. Primers used in this study.

Primer Name	Primer sequence (5'-3')	Descriptions
pET28b_F	CATGGCGCCCTGAAAATACAGGTTTTCGCC	Forward primer to amplify pET28b-p*T7*.
pET28b_R	TAGGGATCCCTAGGGTACCCTAGCGGCCGC	Reverse primer to amplify pET28b-p*T7*.
*bb2925*_F	TGTATTTTCAGGGCGCCatgccgaagaagttttccaatcc	Forward primer to amplify *bb2925* from *B. bronchiseptica* wild type and to be inserted in pET28b-p*T7*.
*bb2925*_R	GGTACCCTAGGGATCCCtacagacgcaggtcggcc	Forward primer to amplify *bb2925* from *B. bronchiseptica* wild type and to be inserted in pET28b-p*T7*.
pCDF_F	CATTAGTAACCTCCTTAATTTTTTTGAGCTCG	Forward primer to amplify pCDF-p*araB*.
pCDF_R	GGTCATCACCATCATCACCACTAAGAGCTCTC	Reverse primer to amplify pCDF-p*araB*.
*bb2924*_F	AATTAAGGAGGTTACTAatgacgacacgatacgaggatttg	Forward primer to amplify *bb2924* from *B. bronchiseptica* wild type and to be inserted in pCDF-p*araB*.
*bb2924*_R	GGTGATGATGGTGATGACCtcgcaggaactgcttgtacc	Forward primer to amplify *bb2924* from *B. bronchiseptica* wild type and to be inserted in pCDF-p*araB*.
*bb2925*_F2	AATTAAGGAGGTTACTAatgccgaagaagttttccaatcc	Forward primer to amplify *bb2925-bb2924* from *B. bronchiseptica* wild type and to be inserted in pCDF-p*araB*, work with reverse primer *bb2924*_R.
*bb0876*_F	TTAAGGAGGTTACTAatggcccccgtgcccgcg	Forward primer to amplify *bb0876-bb0877* from *B. bronchiseptica* wild type and to be inserted in pCDF-p*araB*.
*bb0877*_R	ATGATGGTGATGACCacgcaggaattgcgtgtaccag	Forward primer to amplify *bb0876-bb0877* from *B. bronchiseptica* wild type and to be inserted in pCDF-p*araB*.
pBBR-F	ATTCCGACCAGCGAAGTGAAGTAATCGG	Forward primer to amplify pBBR-pfhaB from the parent vector pBBR-pfhaB-GFP (*57*).
pBBR-R	ACTAGTTCTAGAGCGGCCGCCAC	Reverse primer to amplify pBBR-pfhaB from the parent vector pBBR-pfhaB-GFP
*bb0875*_F	TTCGCTGGTCGGAATcgtctccatagaccaacctAAGGTGac	Forward primer to amplify RBS-*bb0875-bb0879* from *B. bronchiseptica* wild type and to be inserted in pBBR-pfhaB.

*bb0879*_R	CGCTCTAGAACTAGTctact tcgcctggctcagcgcatc	Reverse primer to amplify RBS-*bb0875*-*bb0879* from *B. bronchiseptica* wild type and to be inserted in pBBR-pfhaB.
pBBR-F2	AGAGTCACCTTAGGTTG GTCTATGGAGACG	Forward primer to amplify pBBR-pfhaB-RBS from pBBR-pfhaB-RBS-*bb0875*-*bb0879*, work with reverse primer pBBR-R.
*bb0876*_F2	ACCTAAGGTGACTCTttgcg cattgatgatgtgaaactgg	Forward primer to amplify *bb0876*-*bb0877* and to be inserted in pBBR-pfhaB-RBS.
*bb0877*_R2	CGCTCTAGAACTAGTtcaac gcaggaattgcgtgtaccag	Reverse primer to amplify *bb0876*-*bb0877* and to be inserted in pBBR-pfhaB-RBS.

REFERENCES AND NOTES

1. S. J. Siegel, J. N. Weiser, Mechanisms of bacterial colonization of the respiratory tract. *Annu. Rev. Microbiol.* **69**, 425–444 (2015).

2. A. L. Nelson, A. M. Roche, J. M. Gould, K. Chim, A. J. Ratner, J. N. Weiser, Capsule enhances pneumococcal colonization by limiting mucus-mediated clearance. *Infect. Immun.* **75**, 83–90 (2007).

3. A. D. Magee, J. Yother, Requirement for capsule in colonization by *Streptococcus pneumoniae*. *Infect. Immun.* **69**, 3755–3761 (2001).

4. M. H. Lin, J. C. Shu, L. P. Lin, K. Y. Chong, Y. W. Cheng, J. F. Du, S. T. Liu, Elucidating the crucial role of poly N-acetylglucosamine from *Staphylococcus aureus* in cellular adhesion and pathogenesis. *PLOS ONE* **10**, e0124216 (2015).

5. S. Baur, M. Rautenberg, M. Faulstich, T. Grau, Y. Severin, C. Unger, W. H. Hoffmann, T. Rudel, I. B. Autenrieth, C. Weidenmaier, A nasal epithelial receptor for *Staphylococcus aureus* WTA governs adhesion to epithelial cells and modulates nasal colonization. *PLOS Pathog.* **10**, e1004089 (2014).

6. V. Winstel, P. Kuhner, F. Salomon, J. Larsen, R. Skov, W. Hoffmann, A. Peschel, C. Weidenmaier, Wall teichoic acid glycosylation governs *Staphylococcus aureus* nasal colonization. *mBio* **6**, e00632 (2015).

7. T. Belcher, V. Dubois, A. Rivera-Millot, C. Locht, F. Jacob-Dubuisson, Pathogenicity and virulence of *Bordetella pertussis* and its adaptation to its strictly human host. *Virulence* **12**, 2608–2632 (2021).

8. R. M. Anderson, R. M. May, Directly transmitted infections diseases: Control by vaccination. *Science* **215**, 1053–1060 (1982).

9. R. A. Goodnow, Biology of Bordetella bronchiseptica. *Microbiol. Rev.* **44**, 722–738 (1980).

10. B. S. Schulz, S. Kurz, K. Weber, H. J. Balzer, K. Hartmann, Detection of respiratory viruses and *Bordetella bronchiseptica* in dogs with acute respiratory tract infections. *Vet. J.* **201**, 365–369 (2014).

11. J. M. Warfel, L. I. Zimmerman, T. J. Merkel, Acellular pertussis vaccines protect against disease but fail to prevent infection and transmission in a nonhuman primate model. *Proc. Natl. Acad. Sci. U.S.A.* **111**, 787–792 (2014).

12. J. M. Warfel, L. I. Zimmerman, T. J. Merkel, Comparison of three whole-cell pertussis vaccines in the baboon model of pertussis. *Clin. Vaccine Immunol.* **23**, 47–54 (2016).

13. R. Craig, E. Kunkel, N. S. Crowcroft, M. C. Fitzpatrick, H. de Melker, B. M. Althouse, T. Merkel, S. V. Scarpino, K. Koelle, L. Friedman, C. Arnold, S. Bolotin, Asymptomatic infection and transmission of pertussis in households: A systematic review. *Clin. Infect. Dis.* **70**, 152–161 (2020).

14. A. M. Wendelboe, E. Njamkepo, A. Bourillon, D. D. Floret, J. Gaudelus, M. Gerber, E. Grimprel, D. Greenberg, S. Halperin, J. Liese, F. Muñoz-Rivas, R. Teyssou, N. Guiso, A. Van Rie, Infant Pertussis Study Group Transmission of *Bordetella pertussis* to young infants. *Pediatr. Infect. Dis. J.* **26**, 293–299 (2007),.

15. K. Sekiya, M. Kawahira, Y. Nakase, Protection against experimental *Bordetella bronchiseptica* infection in mice by active immunization with killed vaccine. *Infect. Immun.* **41**, 598–603 (1983).

16. E. T. Harvill, P. A. Cotter, J. F. Miller, Pregenomic comparative analysis between *Bordetella bronchiseptica* RB50 and *Bordetella pertussis* tohama I in murine models of respiratory tract infection. *Infect. Immun.* **67**, 6109–6118, (1999).

17. K. K. Dewan, A. Caulfield, Y. Su, C. J. Sedney, M. Callender, J. Masters, U. Blas-Machado, E. T. Harvill, Adaptive immune protection of the middle ears differs from that of the respiratory tract. *Front. Cell. Infect. Microbiol.* **13**, 1288057 (2023).

18. I. H. Soumana, B. Linz, K. K. Dewan, D. Sarr, M. C. Gestal, L. K. Howard, A. D. Caulfield, B. Rada, E. T. Harvill, Modeling immune evasion and vaccine limitations by targeted nasopharyngeal *Bordetella pertussis* inoculation in mice. *Emerg. Infect. Dis.* **27**, 2107–2116 (2021).

19. Y. Irie, M. H. Yuk, In vivo colonization profile study of *Bordetella bronchiseptica* in the nasal cavity. *FEMS Microbiol. Lett.* **275**, 191–198 (2007).

20. P. A. Cotter, M. H. Yuk, S. Mattoo, B. J. Akerley, J. Boschwitz, D. A. Relman, J. F. Miller, Filamentous hemagglutinin of Bordetella bronchiseptica is required for efficient establishment of tracheal colonization. *Infect. Immun.* **66**, 5921–5929 (1998).

21. T. L. Nicholson, S. L. Brockmeier, C. L. Loving, Contribution of *Bordetella bronchiseptica* filamentous hemagglutinin and pertactin to respiratory disease in swine. *Infect. Immun.* **77**, 2136–2146 (2009).

22. C. S. Inatsuka, S. M. Julio, P. A. Cotter, *Bordetella* filamentous hemagglutinin plays a critical role in immunomodulation, suggesting a mechanism for host specificity. *Proc. Natl. Acad. Sci. U.S.A.* **102**, 18578–18583 (2005).

23. S. Mattoo, J. F. Miller, P. A. Cotter, Role of *Bordetella bronchiseptica* fimbriae in tracheal colonization and development of a humoral immune response. *Infect. Immun.* **68**, 2024–2033 (2000).

24. N. H. Carbonetti, G. V. Artamonova, R. M. Mays, Z. E. Worthington, Pertussis toxin plays an early role in respiratory tract colonization by *Bordetella pertussis*. *Infect. Immun.* **71**, 6358–6366 (2003).

25. T. M. Finn, L. A. Stevens, Tracheal colonization factor: A *Bordetella pertussis* secreted virulence determinant. *Mol. Microbiol.* **16**, 625–634 (1995).

26. M. S. Goodwin, A. A. Weiss, Adenylate cyclase toxin is critical for colonization and pertussis toxin is critical for lethal infection by *Bordetella pertussis* in infant mice. *Infect. Immun.* **58**, 3445–3447 (1990).

27. E. T. Harvill, A. Preston, P. A. Cotter, A. G. Allen, D. J. Maskell, J. F. Miller, Multiple roles for *Bordetella* lipopolysaccharide molecules during respiratory tract infection. *Infect. Immun.* **68**, 6720–6728 (2000).

28. V. C. Burns, E. J. Pishko, A. Preston, D. J. Maskell, E. T. Harvill, Role of *Bordetella* O antigen in respiratory tract infection. *Infect. Immun.* **71**, 86–94 (2003).

29. G. P. Sloan, C. F. Love, N. Sukumar, M. Mishra, R. Deora, The *Bordetella* Bps polysaccharide is critical for biofilm development in the mouse respiratory tract. *J. Bacteriol.* **189**, 8270–8276 (2007).

30. T. Ganguly, J. B. Johnson, N. D. Kock, G. D. Parks, R. Deora, The *Bordetella pertussis* Bps polysaccharide enhances lung colonization by conferring protection from complement-mediated killing. *Cell. Microbiol.* **16**, 1105–1118 (2014).

31. J. Parkhill, M. Sebaihia, A. Preston, L. D. Murphy, N. Thomson, D. E. Harris, M. T. Holden, C. M. Churcher, S. D. Bentley, K. L. Mungall, A. M. Cerdeno-Tarraga, L. Temple, K. James, B. Harris, M. A. Quail, M. Achtman, R. Atkin, S. Baker, D. Basham, N. Bason, I. Cherevach, T. Chillingworth, M. Collins, A. Cronin, P. Davis, J. Doggett, T. Feltwell, A. Goble, N. Hamlin, H. Hauser, S. Holroyd, K. Jagels, S. Leather, S. Moule, H. Norberczak, S. O'Neil, D. Ormond, C. Price, E. Rabbinowitsch, S. Rutter, M. Sanders, D. Saunders, K. Seeger, S. Sharp, M. Simmonds, J. Skelton, R. Squares, S. Squares, K. Stevens, L. Unwin, S. Whitehead, B. G. Barrell, D. J. Maskell, Comparative analysis of the genome sequences of *Bordetella pertussis*, Bordetella parapertussis and *Bordetella bronchiseptica*. *Nat. Genet.* **35**, 32–40 (2003).

32. A. Preston, J. Parkhill, D. J. Maskell, The Bordetellae: Lessons from genomics. *Nat. Rev. Microbiol.* **2**, 379–390 (2004).

33. K. K. Dewan, D. L. Taylor-Mulneix, L. J. Hilburger, I. Rivera, A. Preston, E. T. Harvill, An extracellular polysaccharide locus required for transmission of *Bordetella bronchiseptica*. *J Infect Dis* **216**, 899–906 (2017).

34. T. Yang, Y. Bar-Peled, J. A. Smith, J. Glushka, M. Bar-Peled, In-microbe formation of nucleotide sugars in engineered *Escherichia coli*. *Anal. Biochem.* **421**, 691–698 (2012).

35. R. A. Herbert, K. S. Janardhan, A. R. Pandiri, M. F. Cesta, R. A. Miller, Nose, larynx, and trachea. *Boorman's Pathology of the Rat*, 391–435 (2018).

36. M. Deprez, L. E. Zaragosi, M. Truchi, C. Becavin, S. Ruiz García, M. J. Arguel, M. Plaisant, V. Magnone, K. Lebrigand, S. Abelanet, F. Brau, A. Paquet, D. Pe'er, C. H. Marquette, S. Leroy, P. Barbry, A single-cell atlas of the human healthy airways. *Am. J. Respir. Crit. Care Med.* **202**, 1636–1645 (2020).

37. L. W. Rodenburg, M. Metzemaekers, I. S. van der Windt, S. M. A. Smits, L. A. den Hertog-Oosterhoff, E. Kruisselbrink, J. E. Brunsveld, S. Michel, K. M. de Winter-de Groot, C. K. van der Ent, R. Stadhouders, J. M. Beekman, G. D. Amatngalim, Exploring intrinsic variability between cultured nasal and bronchial epithelia in cystic fibrosis. *Sci. Rep.* **13**, 18573 (2023).

38. H. C. Lam, A. M. Choi, S. W. Ryter, Isolation of mouse respiratory epithelial cells and exposure to experimental cigarette smoke at air liquid interface. *J. Vis. Exp.*, (2011).

39. Y. You, S. L. Brody, Culture and differentiation of mouse tracheal epithelial cells. *Methods Mol. Biol.* **945**, 123–143 (2013).

40. M. B. Antunes, B. A. Woodworth, G. Bhargave, G. Xiong, J. L. Aguilar, A. J. Ratner, J. L. Kreindler, R. C. Rubenstein, N. A. Cohen, Murine nasal septa for respiratory epithelial air-liquid interface cultures. *Biotechniques* **43**, 195–196 (2007).

41. A. M. Floyd, X. Zhou, C. Evans, O. J. Rompala, L. Zhu, M. Wang, Y. Chen, Mucin deficiency causes functional and structural changes of the ocular surface. *PLOS ONE* **7**, e50704 (2012).

42. K. M. Scanlon, Y. G. Snyder, C. Skerry, N. H. Carbonetti, Fatal pertussis in the neonatal mouse model is associated with pertussis toxin-mediated pathology beyond the airways. *Infect. Immun.* **85**, e00355-17 (2017).

43. S. Mattoo, J. D. Cherry, Molecular pathogenesis, epidemiology, and clinical manifestations of respiratory infections due to Bordetella pertussis and other *Bordetella* subspecies. *Clin. Microbiol. Rev.* **18**, 326–382 (2005).

44. E. L. Westman, A. Preston, R. A. Field, J. S. Lam, Biosynthesis of a rare di-N-acetylated sugar in the lipopolysaccharides of both Pseudomonas aeruginosa and Bordetella pertussis occurs via an identical scheme despite different gene clusters. *J. Bacteriol.* **190**, 6060–6069 (2008).

45. B. M. Althouse, S. V. Scarpino, Asymptomatic transmission and the resurgence of *Bordetella pertussis*. *BMC Med.* **13**, 146 (2015).

46. J. R. Harkema, "Chapter 2 - Comparative anatomy and epithelial cell biology of the nose," in *Comparative Biology of the Normal Lung (*ed. 2*),* R. A. Parent, Ed. (Academic Press, 2015), pp. 7–19.

47. J. F. Miller, S. A. Johnson, W. J. Black, D. T. Beattie, J. J. Mekalanos, S. Falkow, Constitutive sensory transduction mutations in the *Bordetella pertussis* bvgS gene. *J. Bacteriol.* **174**, 970–979 (1992).

48. Z. Li, S. Hwang, M. Bar-Peled, Discovery of a unique extracellular polysaccharide in members of the pathogenic *Bacillus* that can co-form with spores. *J. Biol. Chem.* **291**, 19051–19067 (2016).

49. O. Rolin, W. Smallridge, M. Henry, L. Goodfield, D. Place, E. T. Harvill, Toll-like receptor 4 limits transmission of *Bordetella bronchiseptica*. *PLOS ONE* **9**, e85229 (2014).

50. L. Ma, K. K. Dewan, D. L. Taylor-Mulneix, S. M. Wagner, B. Linz, I. Rivera, Y. Su, A. D. Caulfield, U. Blas-Machado, E. T. Harvill, Pertactin contributes to shedding and transmission of *Bordetella bronchiseptica*. *PLOS Pathog.* **17**, e1009735 (2021).

51. S. Z. Hasnain, C. M. Evans, M. Roy, A. L. Gallagher, K. N. Kindrachuk, L. Barron, B. F. Dickey, M. S. Wilson, T. A. Wynn, R. K. Grencis, D. J. Thornton, Muc5ac: A critical component mediating the rejection of enteric nematodes. *J. Exp. Med.* **208**, 893–900 (2011).

52. O. N. Hoang, C. E. Chan, J. M. Brenner, D. Leza-Rincon, A. M. Jaramillo, B. Dolan, A. W. Aziz, R. A. Cardenas, G. J. Cardenas, E. D. Galvez, R. T. Powell, L. Vergara, H. Karmouty-Quintana, J. M. Magnusson, G. C. Hansson, R. Adachi, J. D. Dickinson, C. M. Evans, J. A. Courson, A. R. Burns, M. J. Tuvim, B. F. Dickey, Airway secretory cells contain both a perinuclear golgi ribbon and dispersed golgi satellites. bioRxiv 648270 [Preprint] (2025).

53. T. M. Krunkosky, J. L. Jordan, E. Chambers, D. C. Krause, Mycoplasma pneumoniae host-pathogen studies in an air-liquid culture of differentiated human airway epithelial cells. *Microb. Pathog.* **42**, 98–103 (2007).

54. T. Masuko, A. Minami, N. Iwasaki, T. Majima, S.-I. Nishimura, Y. C. Lee, Carbohydrate analysis by a phenol–sulfuric acid method in microplate format. *Anal. Biochem.* **339**, 69–72 (2005).

55. X. Gu, J. Glushka, S. G. Lee, M. Bar-Peled, Biosynthesis of a new UDP-sugar, UDP-2-acetamido-2-deoxyxylose, in the human pathogen Bacillus cereus subspecies cytotoxis NVH 391-98. *J. Biol. Chem.* **285**, 24825–24833 (2010).

56. P. A. Cotter, J. F. Miller, A mutation in the *Bordetella bronchiseptica* bvgS gene results in reduced virulence and increased resistance to starvation, and identifies a new class of Bvg-regulated antigens. *Mol. Microbiol.* **24**, 671–685 (1997).

57. M. C. Gestal, L. K. Howard, K. Dewan, H. M. Johnson, M. Barbier, C. Bryant, I. H. Soumana, I. Rivera, B. Linz, U. Blas-Machado, E. T. Harvill, Enhancement of immune response against *Bordetella* spp. by disrupting immunomodulation. *Sci. Rep.* **9**, 20261 (2019).
